# Proton-exchange induced reactivity in layered oxides for lithium-ion batteries

**DOI:** 10.1038/s41467-024-53731-2

**Published:** 2024-11-13

**Authors:** Panpan Xu, Xingyu Guo, Binglei Jiao, Jinxing Chen, Minghao Zhang, Haodong Liu, Xiaolu Yu, Maura Appleberry, Zhenzhen Yang, Hongpeng Gao, Fan Yang, Xuefei Weng, Yanbin Shen, Jing Gu, Ying Shirley Meng, Christopher Brooks, Shyue Ping Ong, Zheng Chen

**Affiliations:** 1 Aiiso Yufeng Li Family Department of Chemical and Nano Engineering, University of California, San Diego, La Jolla, CA 92093 USA; 2 Suzhou Institute of Nano-Tech and Nano-Bionics, Chinese Academy of Sciences, 215123 Suzhou, P. R. China; 3 Program of Materials Science and Engineering, University of California, San Diego, La Jolla, CA 92093 USA; 4 Key Laboratory for Computational Physical Sciences (MOE), Institute of Computational Physics, Department of Physics, Fudan University, 200433 Shanghai, China; 5 Institute of Functional Nano & Soft Materials (FUNSOM), Jiangsu Key Laboratory for Carbon-Based Functional Materials & Devices, Soochow University, 215123 Suzhou, Jiangsu P. R. China; 6 Chemical Sciences and Engineering Division, Argonne National Laboratory, 9700 South Cass Avenue, Lemont, IL 60439 USA; 7 Department of Chemistry and Biochemistry, San Diego State University, San Diego, CA USA; 8 Sustainable Power and Energy Center, University of California, San Diego, La Jolla, CA 92093 USA; 9 Honda Research Institute USA, 99P Labs, Columbus, OH 43212 USA; 10 Present Address: Institute of Functional Nano & Soft Materials (FUNSOM), Jiangsu Key Laboratory for Carbon-Based Functional Materials & Devices, Soochow University, 215123 Suzhou, Jiangsu P. R. China

**Keywords:** Batteries, Batteries

## Abstract

LiNi_*x*_Co_*y*_Mn_1-*x*-*y*_O_2_ (0 < *x*, *y* < 1, NCM) is the dominant positive material for the state-of-the-art lithium-ion batteries. However, the sensitivity of NCM materials to moisture makes their manufacturing, storage, transportation, electrode processing and recycling complicated. Although it is recognized that protons play a critical role in their structure stability and performance, proton exchange with Li^+^ in NCM materials has not been well understood. Here, we employ advanced characterizations and computational studies to elucidate how protons intercalate into the layered structure of NCM, leading to the leaching of Li^+^ and the formation of protonated NCM. It is found that protonation facilitates cation rearrangement and formation of impurity phases in NCM, significantly deteriorating structural stability. The adverse effects induced by protons become increasingly pronounced with a higher Ni content in NCM. Through a comprehensive investigation into the thermodynamics and kinetics of protonation, we discover that Li deficiencies in NCM materials can be resolved via solution process in the presence of Li^+^ ions and controlled proton concentration. The underlying mechanism of relithiation is further explored through materials characterizations and kinetics modeling. This work provides crucial insights into controlling structural and compositional defects of Li-ion battery positive material in complicated processing environment.

## Abstract (web summary)

The sensitivity of Li-ion battery materials to moisture complicates their synthesis, storage, processing and recycling. Here, authors show that protonation causes structural instability in LiNi_x_Co_y_Mn_1-x-y_O_2_ (0 <x,y < 1) and that controlled H^+^ and Li^+^ concentration in aqueous processing is critical.

## Introduction

Layered lithium transition metal oxides, also known as NCM (LiNi_*x*_Co_*y*_Mn_1-*x*-*y*_O_2_, where 0 < *x*, *y* < 1), are the primary positive materials for high-energy lithium-ion batteries (LIBs) in use today, from electronic devices to electric vehicles (EVs)^1–3^. The stability of NCM materials, both chemically and structurally, is crucial for optimal performance and battery lifetime. However, exposure to moisture during various stages of the battery life cycle, including manufacturing, storage, electrode processing, and recycling, is often inevitable and can lead to defects and structural degradation^4,5^. For instance, impurities composed of carbonates and hydroxides may form on the surface of NCM particles during ambient storage^6,7^. These defects can lead to reduced electrochemical performance, resulting in capacity loss, poor rate capability, and inferior thermal stability. To mitigate these issues, a water-based washing process is generally employed to remove surface impurities from primarily manufactured NCM particles, but this can cause further loss of active Li^+^ and microphase impurities^8–10^. Alternatively, an approach to enhance structural stability is to coat NCM particles with ceramics such as Al_2_O_3_, LiNbO_3_, and Li_3_PO_4_ using an aqueous solution, which requires careful control of processing conditions to minimize Li^+^ loss-induced cation mixing and phase impurities^11–13^. Additionally, while aqueous electrode processing based on water-soluble binders has been extensively explored to replace toxic and expensive N-Methyl-2-pyrrolidone (NMP)-based coating processes for its environmental and cost benefits, they too have setbacks, such as reduced cycling stability due to similar leaching of active Li^+^ from NMC particles^14–16^.

The degradation of composition and structure mentioned above is a particularly severe problem when it comes to direct recycling of Li-deficient NCM, an emerging approach to recycle LIB-positive materials in which spent positive materials are treated in an aqueous solution to recover their composition^17–20^. While direct recycling has proven effective for many layered oxides electrode materials using an aqueous relithiation process, including LiCoO_2_^17,21^, LiNi_0.33_Co_0.33_Mn_0.33_O_2_ (NCM111)^19^, LiNi_0.5_Co_0.2_Mn_0.3_O_2_ (NC523)^18,19^, and LiNi_0.6_Co_0.2_Mn_0.2_O_2_ (NCM622)^18^, the underlying relithiation mechanism remains unclear. The driving force behind relithiation and key parameters have not been understood, and it is often observed that protons in the aqueous solution interfere with degraded positive materials relithiation, resulting in a loss of Li^+^ and impure phase compositions. A lack of research into the mechanisms of relithiation impedes progress in improving the quality of high-energy positive materials throughout manufacturing, transportation, storage, and recycling.

Here, we focused on examining the thermodynamics and kinetics of proton exchange behavior as well as the resulted compositional and structural defects in NCM materials during aqueous processing. We also studied the impact of these defects on the structural stability, Li^+^ diffusion properties, and electrochemical performance of NCM materials. Our findings indicate that protons exchange with Li^+^ in NCM particles occurs readily in an environment that is rich in H^+^ and devoid of Li^+^. Such an ion exchange phenomenon becomes more pronounced with higher Ni content in NCM materials. Of particular note, a Li^+^ rich solution could promote the lithiation reaction and restore Li deficiencies in NCM materials, while water oxidation occurred simultaneously. Therefore, the concentration of H^+^ and Li^+^ in the solution is critical in maintaining structural stability during aqueous processing. Additionally, the underlying Li^+^/H^+^ exchange mechanism was elucidated via kinetics modeling, density theory function (DFT) calculations, and materials characterization. This work provides a fundamental understanding and effective strategies to mitigate the defects in positive materials induced by protons during manufacturing, functionalization, and recycling in aqueous environment.

## Results

### Competitive mechanism of protonation and relithiation in NCM materials

To systematically compare the proton exchange behavior, we first induced the same concentration of Li deficiencies in NCM111, NCM523, NCM622, and NCM811 through a chemical delithiation method, as described in the Methods^22,23^. These Li-deficient NCM materials are referred as D-NCM. These D-NCM powders were then treated by aqueous solutions in an autoclave at 220 °C with different proton concentrations, where LiOH solutions of 0, 0.1, 1, and 4 M concentration were used (details are described in the experimental section). The treated NCM materials are denoted as W-NCM, 0.1M-NCM, 1M-NCM, and 4M-NCM. As the LiOH concentration increases from 0 to 4 M, the solution pH value rises from 7 to 14.6, while the concentration of protons decreases from 10^−7^ to 2.5 × 10^−15^ M (Figure S1). The concentration difference between H^+^ and Li^+^ in these solutions allows us to systematically investigate the competitive mechanism of these two ions in NCM materials. To investigate the evolution of structural defects and understand the changes in properties during different treatment processes, we used the pristine NCM (P-NCM) as the baseline material for comparison.

**Fig. 1:**
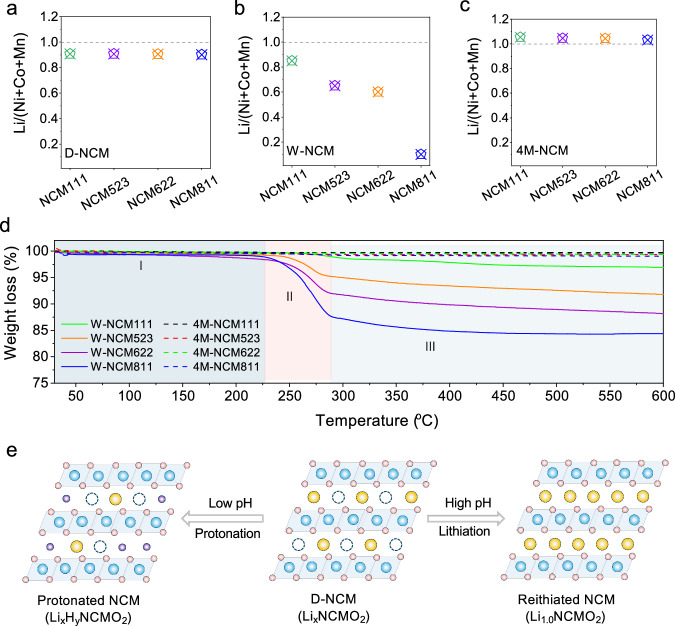
Competitive mechanism of pronation and relithiation of NCM materials. **a**–**c** Li^+^ concentrations in NCM111, 523, 622, and 811 after chemical delithiation (D-NCM), and treatment of pure water (W-NCM) and 4M LiOH solution (4M-NCM). **(d)** TG curves of NCM111, 523, 622, and 811 after treatment of pure water treatment and 4M LiOH solution. **e** Schematic illustration depicting the competitive mechanism of protonation and relithiation in NCM materials. The yellow ball represents Li. The purple ball represents H. The blue ball represents TM. The pink ball represents O. Source data are provided as a Source Data file.

Inductively coupled plasma (ICP) measurement was conducted to assess the Li^+^ content in different NCM materials, which gives the mole ratio of the bulk-Li to transition metals (Ni+Co+Mn). Note that the surface-Li (*i.e*., LiOH, Li_2_CO_3_) on NCM particles can be differentiated by acid-base titration. The results are shown in Fig. S2. Compared to their pristine counterparts, NCM111, 523, 622, and 811 display consistent Li deficiency of approximately 10% after the chemical delithiation process (Fig. 1a). Following the treatment with pure water, the bulk Li^+^ content decreases from 0.9 to 0.82, 0.62, 0.57, and 0.06 for NCM111, 523, 622, and 811 (Fig. 1b), respectively. This has primarily resulted from the exchange behavior of H^+^ and Li^+^, as evidenced by the time-of-flight secondary ion mass spectrometry (TOF-SIMS) results in Fig. S3. Additionally, while NCM111 is often considered stable against aqueous processing, approximately 5% of Li^+^ still leaches into water, a figure that surged to nearly 80% for NCM811. In contrast, when LiOH solutions was used, especially with 4M LiOH, Li^+^ content in all the NCM materials can recover to around 1.0 (Figs. 1c and S4). The loss of Li^+^ from the NCM particles when treated with pure water is hypothesized to be induced by abundant H^+^ in water, facilitating the exchange with Li^+^. This H^+^/Li^+^ exchange behavior is suppressed in LiOH solutions since the concentration of H^+^ in the solutions dramatically decreases.

Thermogravimetric analysis (TGA) was conducted to investigate the presence of H^+^ in the NCM materials treated with pure water (Fig. 1d). Besides the weight loss due to physically adsorbed water (region I), a noticeable weight loss between 230 °C and 280 °C (region II) is observed for all the W-NCM materials. This loss is attributed to the removal of intercalated H^+^ in the form of H_2_O during heating^9,10,24^. Particularly, the weight loss in this region for NCM811 is substantially higher than that for NCM111 (15% vs. 1%), suggesting a larger amount of H^+^ present in the NCM811 particles. At temperature above 280 °C, all the W-NCM materials display multiple stages of weight loss, which can be ascribed to the phase transformation of layered structure to spinel and rock salt phases accompanied by O_2_ loss^25,26^. In contrast, when LiOH is introduced into the solutions, the weight loss associated with H^+^ disappears, and the thermal stability of the treated materials is comparable to that of pristine ones^27,28^, indicating the effective elimination of H^+^ intercalation and successful repair of Li vacancies during the treatment process.

The competitive intercalation of H^+^ and Li^+^ into the layered structure was then dissected through cyclic voltammetry (CV) tests, where different NCM materials served as the working electrodes, a graphite plate as the counter electrode and Ag/AgCl as the reference electrode. 2M Li_2_SO_4_ and 4M LiOH solutions were used as the electrolytes. As shown in Fig. S5a–d, all the NCM materials are electrochemically unstable in the Li_2_SO_4_ electrolyte. An evident pair of redox peaks associated with Li^+^ intercalation/deintercalation was observed in the first cycle. These peaks progressively weakened and broadened, particularly for NCM811. This could be due to H^+^ intercalation into the NCM after Li^+^ deintercalation, which leads to poor electrochemical kinetics and reversibility^29,30^. It is worth noting that significant H^+^ intercalation still occurs despite a high Li^+^ concentration in the 2M Li_2_SO_4_ electrolyte, indicating that the H^+^ concentration dictates the H^+^/Li^+^ exchange in aqueous solutions.

We then employed a 4M LiOH solution as the electrolyte, which can reduce the H^+^ concentration by eight orders of magnitude. The CV curves of all the NCM materials display one pair of peaks associated with Li^+^ intercalation and deintercalation and these peaks stabilize after the first cycle (Fig. S5e–h), indicative of a substantial improvement in the stability and reversibility of the NCM materials in this alkaline electrolyte. This observation is consistent with the previous reports about CV stability of NCM electrodes in high-pH electrolytes^29,31^. However, the O_2_ evolution potential at high pH level begins to overlap with the Li^+^ intercalation/deintercalation potential, which would limit the full intercalation of Li^+^ in the NCM.

The elimination of H^+^ interaction in alkaline electrolytes can be explained by the Nernst equation as follows:

1 $${{Li}}_{x}{TM}{O}_{2}\left(x \, < \,1\right)+y{H}^{+}+{{ye}}^{-}\to {{Li}}_{x}{H}_{y}{TM}{O}_{2}$$

2 $${{Li}}_{x}{TM}{O}_{2}\left(x \, < \, 1\right)+(1-x){{Li}}^{+}+(1-x){e}^{-}\to {{Li}}_{1.0}{TM}{O}_{2}$$

3 $${E}_{{H}^{+}}={{E}_{{H}^{+}}}^{{{\theta }}}+\frac{{{RT}}}{{{zF}}}{{ln}}\frac{{\left({{{\alpha }}}_{{{H}}}+\right)}^{{{y}}}\left({{\alpha }}{{{Li}}}_{{{X}}}{{{TOM}}}_{2}\right)}{{{\alpha }}{{{Li}}}_{{{\rm{X}}}}{{{H}}}_{{{y}}}{{{TOM}}}_{2}}$$

4 $${E}_{{{Li}}^{+}}={{E}_{{{Li}}^{+}}}^{{{\theta }}}+\frac{{RT}}{{zF}}{In}\frac{{\left({{{\alpha }}}_{{{\rm{Li}}}}+\right)}^{1-{{x}}}\left({{\alpha }}{{{Li}}}_{{{X}}}{{{TOM}}}_{2}\right)}{{{\alpha }}{{{Li}}}_{1.0}{{{TOM}}}_{2}}$$

5 $$\Delta G=-{nFE}$$

where *E* and *E*^* θ*^ are the practical and standard potentials for H^+^ intercalation reaction, *R* represents the gas constant, *T* is temperature, *z* is the number of electrons participating in the reaction, *F* refers to the Faraday constant, and *α* is the concentration of different species. Through inspection of the Nernst equation and comparing with CV results, the H^+^ and Li^+^ intercalation potentials are proportional to their concentrations. Specifically, with the increase of solution pH values from 7 to 14.6, the concentration of H^+^ can decrease from 10^−7^ to 2.5 × 10^−15^ M (Figure S1). The intercalation energy for H^+^ increases from −23.16 to 20.25 kJ mol^−1^ when 4M LiOH solution is used, meaning this reaction is thermodynamically unfavorable in the 4 M LiOH solution (Figure S6). Thus, a high pH value in aqueous solutions is critical for suppressing the protonation of NCM materials. Additionally, we also evaluated the dependence of Li^+^ intercalation barrier on the concentration of Li^+^ and found that this barrier is reduced from −4.63 to −13.78 kJ mol^−1^ as the LiOH concentration increases from 0.1 to 4 M. This suggests that a high concentration of Li^+^ could effectively facilitate the lithiation process. Thus, concentrated LiOH can well maintain the structural stability of NCM materials in aqueous solutions.

Overall, our findings indicate that protons in aqueous solutions readily exchange with Li^+^ in NCM, leading to a pronounced protonation reaction, especially for those with higher Ni content (Fig. 1e). However, this protonation behavior can be effectively eliminated by employing concentrated LiOH solutions. Furthermore, these solutions can facilitate the diffusion of Li^+^ in the solution into the Li vacancies within NCM particles, thereby completing the relithiation process.

**Fig. 2:**
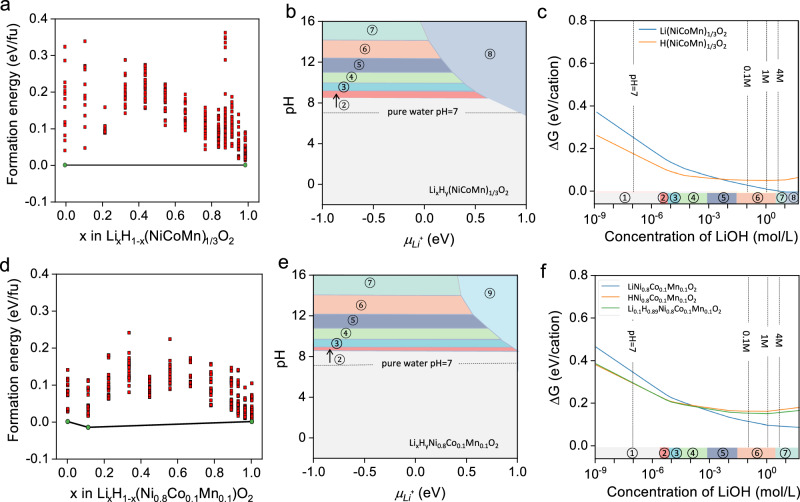
Phase stabilities of protonated and relithiated NCM materials. **a** Calculated binary phase diagram of protonation of (**c**) Li_x_H_1-x_(NiCoMn)O_2_. **b** Calculated phase diagram of Li_*x*_H_*y*_(NiCoMn)_1/3_O_2_ (0 ≤ *x*,*y* ≤ 1) as a function of pH and ${\mu }_{{{{\rm{Li}}}}^{+}}$. Regions with solid phases are shaded in lake blue. **c** Changes of ∆*G* of Li(NiCoMn)_1/3_O_2_ as a function of concentration of LiOH solution. **d** Calculated binary phase diagram of protonation of Li_*x*_H_1-*x*_(Ni_0.8_Co_0.1_Mn_0.1_)O_2_ (*x* = 0 ~ 1.0). **e** Calculated phase diagram of Li_*x*_H_*y*_Ni_0.8_Co_0.1_Mn_0.1_O_2_ (0 ≤ *x*,*y* ≤ 1) as a function of pH and ${\mu }_{{{{\rm{Li}}}}^{+}}$. Regions with solid phases are shaded in lake blue. **f** Changes of ∆*G* of LiNi_0.8_Co_0.1_Mn_0.1_O_2_, HNi_0.8_Co_0.1_Mn_0.1_O_2_ and Li_0.11_H_0.89_Ni_0.8_Co_0.1_Mn_0.1_O_2_ as a function of concentration of LiOH solution. A material that is thermodynamically stable under certain conditions in an aqueous environment has ∆*G* = 0 eV/cation. A larger ∆*G* indicates higher possibility of a material to decompose into combinations of stable species indicated on phase diagram. Based on previous studies by Singh et al., materials with ∆*G* < 0.5 eV/cation is able to resist dissolution, particularly when bulk solid-state transformations are involved^18^. The phase of NCM in region ① presents Ni^2+^, Co^2+^ and Mn^2+^; region ② presents Co(OH)_2_(s), Ni^2+^and Mn^2+^; region ③ presents Co(OH)_2_, Ni(OH)_2_ and Mn^2+^; region ④ presents Co(OH)_2_, Ni(OH)_2_ and MnHO_2_; region ⑤ presents Co_3_O_4_, Ni(OH)_2_ and MnHO_2_; region⑥ presents region Co_3_O_4_, Ni(OH)_3_^-^ and MnHO_2_; region ⑦ presents Co_3_O_4_, Ni(OH)_4_^2-^ and MnHO_2_; region ⑧ presents Li(NiCoMn)_1/3_O_2_; region ⑨ presents LiNi_0.8_Co_0.1_Mn_0.1_O_2_. Source data are provided as a Source Data file.

We then conducted DFT calculations to investigate the formation energies of NCM materials with different content of Li^+^ and H^+^ (Figure S7, Fig. 2a, b). The details of calculation were described in supplementary information. In NCM111, the partially protonated structures are predicted to be unstable with positive formation energies. For NCM811 structures, only Li_0.11_H_0.89_(Ni_0.8_Co_0.1_Mn_0.1_)O_2_ lies on the convex hull with a small negative formation energy of -0.015 eV/fu, while other structures are predicted to be unstable. These results are in line with previous studies^32^ that showed mixing protons and lithium is thermodynamically unfavorable due to the differing stacking preferences of Li and H in layered oxide structures (O3 *vs*. P3, respectively).

In aqueous environments, the relative stabilities of lithiated and protonated NCM, as measured by the Gibbs free energy relative the most stable decomposition products ∆*G*, exhibit a strong dependence on the concentration of LiOH. At neutral pH (pH = 7), protonated NCM111 are more stable than lithiated NCM111 (Fig. 2c, e). However, increasing the concentration of LiOH increases the relative stability of lithiated NCM over protonated NCM. At LiOH concentration > 0.1M, the lithiated NCM is more stable than the protonated NCM. At LiOH concentrations greater than ~4 M, lithiated NCM111 becomes thermodynamically stable against decomposition. We note that, in general, both the lithiated and protonated phases exhibit good aqueous stability with ∆*G* < 0.5 eV/cation, especially, when the concentration of LiOH is > 10^−5^ M. Furthermore, higher LiOH concentrations are associated with decomposition reactions with solid products, which may help passivate against further reactions^33–35^. Similar trends are observed for NCM811 (Fig. 2d, f), though the ∆*G* of lithiated and protonated NCM811 never becomes negative as in the case of NCM111, i.e., NCM811 has a poorer aqueous stability than NCM111, especially in lithium dilute and less basic solutions. These results are in line with the experimental observations of enhanced relithiation in D-NCM when treated with LiOH solutions at 0.1, 1 and 4 M and significant lithium loss in D-NCM treated by pure water.

It is worth noting that despite the fully protonated NCM compound is predicted to be thermodynamically favored, they have not been observed in hydrothermal experiments in this work. These deviations are likely due to the difficulty in mixing protons and lithium in the same structure. This can be seen from the high formation energies of the partially protonated NCM phases shown in Fig. 2a, b (Supplementary Data 1 and 2). At the same time, the H^+^/Li^+^ exchange reaction can be sluggish due to the repulsion of Li^+^ and H^+^ ions^32^ and the increased Li^+^ migration barrier caused by protonation. When cycling NCM materials in 2M Li_2_SO_4_ electrolyte within voltage ranges of 0 to 1.2 V, the redox peaks associated with Li^+^ (de−) intercalation are progressively weakened and broadened from the 1^st^ to the 3^rd^ cycles (Figure S5). This result indicates an irreversible deintercalation of Li^+^ in neutral solutions. As our calculations indicate that the protonated phase is thermodynamically more stable in neutral solution, we suggest that full protonation may occur after the full de-lithiation of NCM. In addition, when treated with pure water, a greater Li^+^ loss is observed in NCM with higher Ni content. This can be attributed to their increased aqueous instability (higher ∆*G*) in LiOH solutions and lower formation energies of partially protonated NCM relative to fully lithiated and protonated counterparts.

### Crystal lattice evolution in NCM induced by protons

Given the potential adverse effects of protons on the performance and safety of NCM materials, it is imperative to gain a comprehensive understanding of the morphological, structural, and compositional changes induced by proton interactions. The morphologies of D-NCM samples, treated with both pure water and a 4 M LiOH solution, were meticulously characterized using scanning electron microscopy (SEM). It is found that the aqueous treatment of NCM materials did not affect their overall morphology (Fig. S8a–p).

**Fig. 3:**
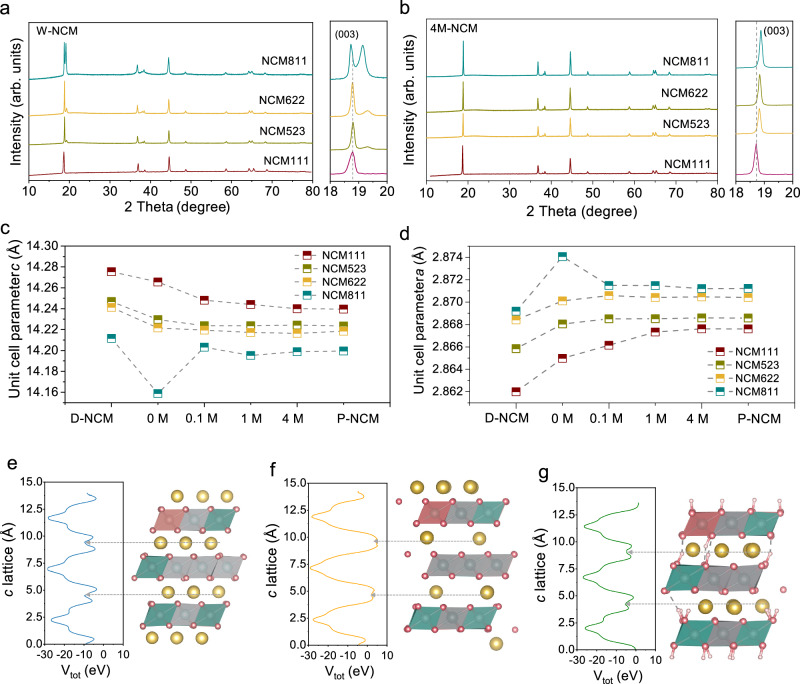
Crystal lattice evolution induced by protons. **a**, **b** XRD patterns of pure water and 4M LiOH solution treated NCM materials (W-NCM and 4M-NCM). **c**, **d** *c* and *a* lattice parameter evolution of NCM materials after treatment of chemical delithiation, pure water, 0.1M, 1M and 4M LiOH solutions. **e**–**g** Relaxed structures and calculated planar-average (along [001]) of the total potential energy of Li(NiCoMn)_1/3_O_2_ (**e**), Li_0.44_(NiCoMn)_1/3_O_2_ (**f**) and Li_0.44_H_0.54_(NiCoMn)_1/3_O_2_ (**g**). The yellow ball represents Li ions. The pink octahedral represents CoO_6_. The gray octahedral represents MnO_6._ The blue octahedral represents NiO_6_. Source data are provided as a Source Data file.

The phase characteristics of the resulting materials were assessed using X-ray powder diffraction (XRD, Figs. S9–S12). The XRD results of NCM samples treated with pure water and 4M LiOH solution are compared in Fig. 3a, b. The main peaks of all the W-NCM materials maintain *α*-NaFeO_2_ structure with rhombohedral $R\bar{3}m$ space group, indicating the preservation of the bulk layered structure (Fig. 3a). Intriguingly, the (003) peak of the materials treated with pure water (0M LiOH) shifts to low diffraction angles, implying loss of Li^+^ in NCM particles. Moreover, aside from the NCM111 sample, a new diffraction peak at 19.2^o^, associated with the protonated NCM phase emerged in the XRD profiles of W-NCM523, 622, and 811^36,37^. The intensity of this peak increases with the increase of the Ni content. This observation suggests that the NCM with higher Ni content is more vulnerable towards H^+^ replacement. In contrast, when the samples were treated with a 4M LiOH solution, all NCM materials exhibited well-defined layered structures like pristine samples (Fig. 3b).

The lattice parameters of NCM materials treated by different concentrations of LiOH solutions were examined by using XRD Rietveld refinement analysis (Figs. S13–S16, Table S1) and their evolution after treatment with different solutions is summarized in Fig. 3c, d. Compared to P-NCM materials, the extraction of 10% of Li^+^ (D-NCM) led to an increased *c* and reduced *a* lattice parameter^38,39^. Upon treatment of D-NCM with pure water, all NCM materials exhibited a reduction in the *c* lattice parameter and an increment in the *a* lattice parameter due to the incorporation of protons^8^.

The *c* lattice parameters of both NCM111 and NCM811 first expand and then contract when more than 0.56 Li is deintercalated (Fig. S17, Supplementary Data 1 and 2). The expansion of the *c*-axis during the de-lithiation can be attributed to the reduced screening effect from Li^+^, which increases the repulsion between adjacent oxygen planes. The subsequent shrinkage of the *c*-axis is likely due to the combined effects of the dissipation of effective charge on oxygen and residual nonlocal dispersion forces from Li^+^ vacancies^40,41^. The deintercalation of Li^+^ is accompanied by the oxidation of Ni from Ni^2+^ to Ni^3+^/Ni^4+^. These observations are supported by the DFT calculations. As shown in Fig. 3e, f, compared to Li(NiCoMn)_1/3_O_2_, the dips in the total potential energy *V*_tot_ averaged along the *c*-axis for Li_0.44_(NiCoMn)_1/3_O_2_ increase, indicating charge transferred from Li layers back to the oxygen layers upon Li^+^ removal. In contrast, the *c* lattice parameters slightly decrease as more Li^+^ is deintercalated and protons are inserted simultaneously. This is supported by the observation that there is little charge transfer between Li atoms and adjacent oxygen ions when protons are inserted (Fig. 3g). The slight decrease of *c* lattice during protonation might be attributed to the smaller size of H^+^ compared with Li^+^.

During the de-lithiation process, the *a* lattice parameters of both NCM materials decrease continuously. The average Ni–O bond length in Li_0.44_(NiCoMn)_1/3_O_2_ contracts from 2.05 to 1.96 Å, whereas the Mn–O and Co–O bond lengths remain relatively stable. This contraction of *a* lattice during de-lithiation can be primarily ascribed to the oxidation of Ni^2+^ to Ni^3+^. In contrast, protonation leads to an expansion of the *a* lattice parameter. The transition metal to oxygen (M–O) bond lengths in protonated NCM are extended due to the presence of inserted protons. Especially, in layered oxides, the DFT-calculated average Ni-O bond length of NiO_6_ complexes is about 2.0 Å, while the calculated average Ni-O bond length of Ni(OH)_6_ complex is about 1.91 Å. Thus, protonation diminishes the electrostatic interaction between the transition metal ions and oxygen, resulting in an increase in the *a* lattice parameter.

It is noteworthy to mention that W-NCM811 exhibits a sharp decrease in *c* lattice parameter and an increase in *a* lattice parameter, suggesting the high degree of protonation in this material has severely disrupted the crystal structure. We then compared the experimentally measured lattice parameters of pure water and LiOH-treated NCM111 and NCM811 with the calculated lattice parameters of structures with equal Li^+^ amount (Fig. S18). We found that the protonated NCM811 displays a similar evolution trend to that of the experimental observation.

When LiOH solutions were utilized, although all the main XRD peaks for the treated samples appeared identical to the P-NCM, variations in the crystal structure were detected. Specifically, with 0.1 M LiOH, a slight increment in the c lattice parameter and a decrement in a lattice parameter were noted compared with D-NCM, indicating Li^+^ intercalation into the vacancies. As the LiOH concentration increased from 0.1M to 4M, the *c* and *a* lattice parameters progressively increased until they approached the values of the P-NCM. The above results further confirm the protonation of D-NCM in pure water and enhanced re-lithiation in more concentrated LiOH solutions, which is consistent the thermodynamics calculation.

### Surface degradation in NCM induced by protons

**Fig. 4:**
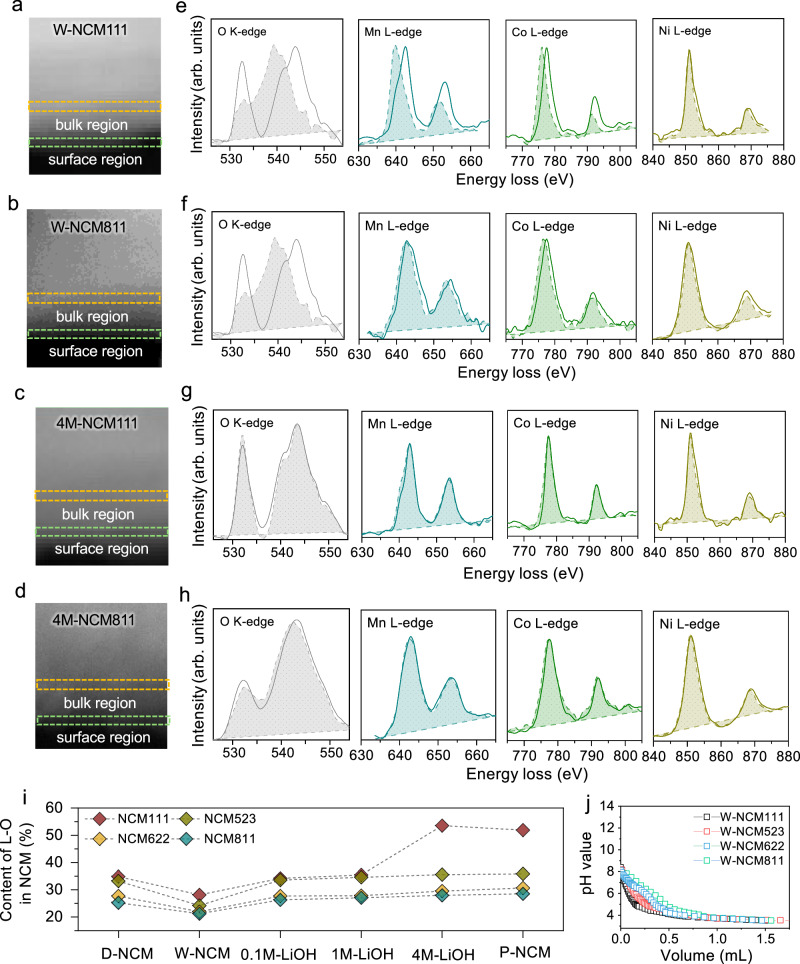
Surface degradation induced by protons. **a**–**d** HAADF-STEM images of NCM111 and NCM811 materials treated with pure water (W-NCM) and 4M LiOH solution (4M-NCM). **e**–**h** Spatially resolved EELS spectra from the bulk to the surface of NCM111 and NCM811 materials treated with pure water and 4M LiOH solution. In (**a**–**d**), the green dashed box highlights the surface region of the materials, while the orange dashed box indicates the bulk region. The EELS spectra obtained from the surface regions are shown as solid lines, whereas spectra from the bulk regions are depicted as dashed lines with shaded regions. **i** Content of L-O in D-NCM and P-NCM, as well as the resulting materials after treatment with pure water, 0.1M, 1M, and 4M LiOH solutions. **j** Titration curves of W-NCM111, 523, 622 and 811. Source data are provided as a Source Data file.

To gain deeper insights into proton-induced structural defects, high-angle annular dark-field scanning transmission electron microscopic (HAADF-STEM) combined with electron energy loss spectroscopy (EELS) was performed. The EELS spectra were obtained from both a surface region (green box) and a bulk region (orange box) in NCM particles, as indicated in the STEM images (Figs. 4a–d, S19–20). The bulk region is located approximately 10 nm beneath the surface. For the O K-edge EELS spectra (Fig. 4e, f), a pre-peak at 532 eV and a main peak at 540 eV were observed in the W-NCM particle bulk, which usually corresponds to the splitting of the TM *3d* orbitals in a six-coordinated environment. The high intensity of the pre-peak is usually indicative of a well-defined layered structure^42,43^. Upon water treatment, the pre-peak in the O K-edge EELS spectra on the surface of W-NCM materials notably weakened. The main peak of the O K-edge shifted to lower positions as the EELS acquisition moved closer to the particle surface. These changes suggest a reduction in neighboring TM ions around O atoms, resulting in an alteration of the splitting of adjacent TM *3d* orbitals from a typical six-coordinated configuration^44^. The reduction of TM ions was further evident in the TM L-edge spectra.

To gain further insights into the phase information, we conducted high resolution transmission electron microscope (HRTEM) and fast Fourier transformation (FFT) transformation. The W-NCM111 present layered structure in the bulk region, but spinel phase was observed in the surface region (Fig. S21a). This phase transformation might be resulted from the proton-induced migration of Mn ions^45^. While for W-NCM811(Fig. S21b), the surface and bulk regions were found to be layered structure but with complicated lattice planes, which might come from the substitution of Li^+^ by H^+^, resulting in lattice distortion. Considering approximately 80% of Li^+^ have been replaced by H^+^, the surface-to-bulk structure likely corresponds to HTMO_2_^8^. Consequently, no significant shift in EELS spectra of transition metals is observed for the transition metals in W-NCM811 from the surface to a depth of 10 nm.

Unlike the surface degradation observed in W-NCM materials, all the NCM treated with 4M LiOH exhibit a stable lattice structure compared with that of W-NCM samples, suggested by negligible changes of intensity and position of the pre-peak in the O K-edge, as well as Mn, Co, and Ni L-edge for both the surface and bulk regions (Fig. 4g, h). Therefore, LiOH solution treatment effectively mitigates the adverse effects of H^+^ on surface structure alterations.

To investigate the evolution of surface composition induced by protons, X-ray photoelectron spectroscopy (XPS) was conducted. The high-resolution O *1s* XPS spectra of all the samples could be fitted into three peaks (Fig. S22). The primary peak at 529.5 eV corresponds to bulk lattice oxygen (bulk L–O)^44^. Another peak at approximately 531.5 eV is assigned to -OH and CO_3_^2-^ in literatures^46–48^. The third peak, at around 533 eV, is primarily associated with adsorbed H_2_O/CO_2_, referred to as “ads. O”^46,49^. With pure water treatment, the bulk L-O peak showed a noticeable decrease, accompanied by an increase in the peak associated with non-bulk L–O. Furthermore, as the Ni content in NCM increases, the peak of non-bulk L–O significantly increased. However, with a LiOH solution treatment, the bulk L–O peak gradually increases with the increase of the LiOH concentration. Notably, the samples treated with 4M LiOH exhibit almost identical intensity of the bulk L–O peaks to that of pristine materials.

We further conducted a quantitative analysis of the content of bulk L–O in the treated NCM materials (Fig. 4i). With pure water treatment, the content of bulk L–O decreases from 35%, 33%, 27% and 25% to 28%, 24%, 22% and 21% as the Ni content increases from 0.33 to 0.5, 0.6 and 0.8 in NCM. Considering the low volume of HCl consumed for titration, impurities like LiOH and Li_2_CO_3_ on the surface of W-NCM materials can be ignored (Fig. 4j). The reduced content of bulk L–O is mainly caused by the replacement of Li in LiO_6_ structure by H^+^, leading to the formation of –OH. Greater replacement is observed with the increase of Ni in NCM. However, the bulk L–O content of the treated NCM rapidly decreases with the LiOH concentration increases from 0.1M to 1M, reaching similar values of the pristine samples after 4M LiOH treatment. Such an improvement is attributed to the suppression of H^+^ replacement and restoration of the Li^+^ composition of NCM in LiOH solutions.

To understand the impact of H^+^ replacement on NCM surface degradation, we investigated the interaction of W-NCM811 and 4M-NCM811 with CO_2_ (Fig. S23a) using near-ambient pressure X-ray photoelectron spectroscopy (NAP-XPS). A thorough surface cleaning step employing Ar^+^ etching was conducted to clean the surface for these samples to ensure no C-related signals (Fig. S23b, c) before CO_2_ introduction. The O *1s* spectrum of 4M-NCM811 after surface cleaning displayed a primary peak and a small shoulder peak, identical to that of commercial NCM811 (Fig. S23d). In contrast, besides the primary L-O peak, an additional peak at 530.5 eV emerged, attributed to -OOH in the W-NCM811(Fig. S23e)^50^, which arises from the bonding of intercalated H^+^ with O in the TMO_6_ octahedral structure^51–54^. This observation aligns with the new phase associated with oxyhydroxide observed in the XRD patterns (Fig. 3a). Upon introducing CO_2_, W-NCM811 displayed a stronger peak related to adsorbed CO_2_ in both the C *1s* and O *1s* spectra compared to 4M-NCM811, indicating more CO_2_ adsorption facilitated by protons in NCM.

### Degradation of Li^+^ diffusion kinetics in NCM induced by protons

To investigate any potential electrochemical adverse effects, we conducted online electrochemical mass spectrometry (OEMS) to monitor gas generation during cell charging at a low rate of 0.1C (Fig. S24). To specifically examine the influence of H^+^ on battery performance, we chose to set the cutoff voltage at 4.3 V during the electrochemical testing.

**Fig. 5:**
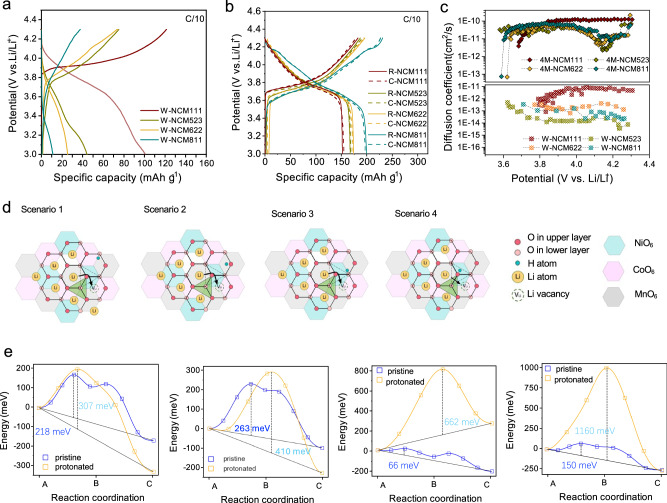
Degradation of Li^+^ diffusion kinetics induced by protons. **a**, **b** The voltage profiles of protonated (W-NCM) and relithiated (R-NCM) materials, as well as commercial materials (C-NCM) in the voltage range of 3-4.3 V. **c** deduced diffusion coefficients of protonated and relithiated materials. **d** The pathway of Li^+^ migration in protonated NCM111. **e** Selected calculated Li^+^ migration barrier in pristine and protonated NCM111 with proton absorbed onto different O atoms. Source data are provided as a Source Data file.

The charge/discharge profiles at a rate of 0.1 C (1C corresponds150, 170, 180, 200 mA/g for NCM111, 523, 622, and 811, respectively) for the NCM materials before and after delithiation are presented in Figure S25. The extraction of 10% of Li^+^ in NCM111, 523, 622, and 811 reduced the discharge capacity to 143, 155, 160, and 178 mAh/g, respectively, accounting for nearly 90% of the capacity of the pristine materials. However, after treatment with pure water, the resulting NCM111, 523, 622, and 811 materials exhibited capacities of only 99, 44, 25, and 11 mAh/g, respectively (Fig. 5a). These values are significantly lower than those of the D-NCM materials, indicating a further loss of Li^+^ during the pure water treatment, especially for NCM811. The results are consistent with those obtained from DFT calculations (Fig. S26), which show that protonated Li_0.85_H_0.15_(NiCoMn)_1/3_O_2_ experience a capacity loss of 50 mAh/g compared to their fully lithiated counterparts. The protonated Li_0.1_H_0.9_Ni_0.78_Co_0.11_Mn_0.11_O_2_ experience a much larger capacity loss of 155 mAh/g. In contrast, when 4M LiOH solutions are used, the capacities can be restored to the same level as the pristine materials (Fig. 5b).

The electrochemical kinetics of pronated and relithiated NCM materials was then studied by the galvanostatic intermittent titration technique (GITT) test. The Li^+^ diffusion coefficient $({D}_{{{Li}}^{+}})$ is determined based on Eq. (6)^55^:

6 $${{D}}_{{{Li}}^{+}}=\frac{{4{\mbox{r}}}^{2}}{\pi\tau}{\left(\frac{\Delta {{{E}}}_{{{s}}}}{{\Delta {{E}}}_{\tau }}\right)}^{2}$$

where *r* is the particle radius of the positive electrode material, *τ* is the short pulse time, Δ*E*_*s*_ is the change in steady-state voltage, and Δ*E*_*τ*_ is the change in transient voltage^56^. The W- NCM111 exhibits a relatively high ${D}_{{{Li}}^{+}}$ of ~7 × 10^−12^ cm^2^ s^−1^ because of a relatively low concentration of protons within the particles (Fig. 5c). However, for NCM with higher Ni content, ${D}_{{{Li}}^{+}}$ is reduced by one to two orders of magnitude. This reduction is primarily attributed to the presence of more H^+^ in the lattice, which increases the Li^+^ diffusion barrier. In contrast, the 4M-NCM materials delivered much improved Li^+^ diffusion kinetics. As shown in Fig. S27, in layered oxides, Li^+^ migrates via “di-vacancy” mechanism with low migration energy barriers ranging from 66 to 262 meV^57^, indicating facile Li^+^ diffusivities. When a proton is absorbed by the oxygen atom closest to the metastable tetrahedral site (Fig. 5d, e), the Li^+^ migration barrier in protonated NCM can be ~1000 meV higher than those in pristine NCM materials. When the proton attaches to oxygen atoms next nearest to the tetrahedral site, the energy increase is smaller but still relatively large (~600 meV). As the proton binds to oxygen atoms further away from the tetrahedral sites, the increment decreases, ranging from 100 to 150 meV.

### Mechanism of direct regeneration based on hydrothermal relithiation

To figure out the important roles of Li^+^ and OH^-^ in the relithiation process, we also used 2M Li_2_SO_4_ solution to treat D-NCM, which maintains almost the same concentration of H^+^ while providing a Li^+^ rich environment comparable with 4M LiOH. However, this sample only delivers a capacity similar to that of W-NCM (Fig. S28), suggesting the failure of re-lithiation. This result indicates that the relithiation step strongly depends on the OH^-^ concentration rather than just Li^+^ concentration.

**Fig. 6:**
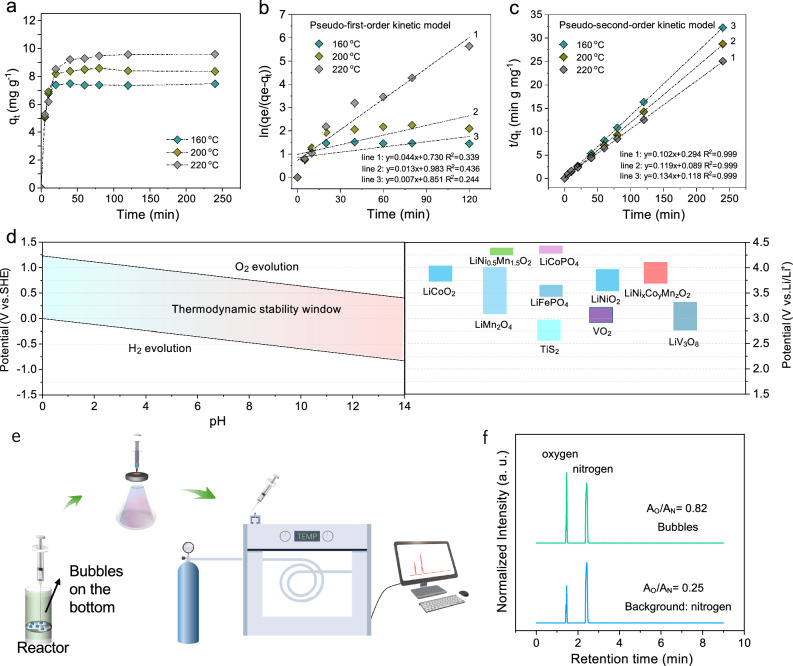
Underlying mechanism of hydrothermal relithiation. **a** Adsorption kinetic curves of the hydrothermal relithiation at different temperatures. **b** Pseudo-first-order kinetic model. **c** Pseudo-first-order kinetic model. **d** Pourbaix diagram showing the thermodynamic stability window of water and Li ions intercalation potential of various positive electrode materials. **e** Schematic illustration of GC measurement. **f** GC chromatogram of the background gas and gas in bubbles. Source data are provided as a Source Data file.

Furthermore, we studied kinetics involved in the repairing Li-deficient NCM materials by chemical relithiation in LiOH solutions. The amount of Li^+^ adsorbed by NCM at different time intervals was recorded (Fig. 6a). Due to the fast Li^+^ diffusion in the Li-rich liquid environment, the relithiation was almost completed in 60 min for different temperatures, except for the minor difference in the total absorption capacity at equilibrium state. The reactions were modeled with pseudo-first-order kinetic model (Eq. 7) or pseudo-second-order kinetic model (Eq. 8):

7 $${\mathrm{ln}}\left(\frac{{q}_{e}}{{q}_{e}-{q}_{t}}\right)={k}_{1}t$$

8 $$\frac{t}{{q}_{t}}=\frac{1}{{k}_{2}{{q}_{e}}^{2}}+\frac{t}{{q}_{e}}$$

where *q*_*e*_ is the amount of Li^+^ adsorbed per gram of the degraded positive material at equilibrium (mg g^−1^), *q*_*t*_ is the amount of Li^+^ adsorbed per gram of the degraded positive material (mg g^−1^) at time t (min), *k*_*1*_ is the pseudo-first-order rate constant (min^−1^), and *k*_*2*_ is the pseudo-second-order rate constant (g mg^−1^ min^−1^). The fitting results are shown in Fig. 6b, c. Obviously, the linearity of the pseudo-second model is much better than that of the pseudo-first model, which showed a coefficient of determination of 0.999. Thus, the relithiation is found to be controlled by chemisorption, rather than physisorption, meaning that valence forces by sharing or exchanging electrons between NCM and LiOH is involved. This is similar to typical electrochemical discharge process, where the intercalation of Li^+^ ions is accompanied by reduction of transition metals (TMs)^53^.

Considering the relithiation system studied here, OH^-^ is the only possible substance that may play the role of donating electrons. We compared the Pourbaix diagram^54^ of water and Li^+^ ions intercalation potential of various positive materials. It is obvious that the oxidation potential of water decreases with the increase of pH value (Fig. 6d). The Li-bearing solution for relithiation shows strong alkalinity, leading to lower oxidation potential than the reduction potential of NCM materials. Hence, water could be oxidized by transition metals in NCM materials and release O_2_^58^_._ Experimentally, after treatment with 4M LiOH, some small bubbles at the bottom of the reactor were observed (Fig. S29). The gas in the bubble was transferred with a syringe to a bottle full of N_2_ to be analyzed with gas chromatography (GC) (Fig. 6e). The relative integrated area of O_2_ and N_2_ of the collected sample increased from 0.52 (background) to 0.82 (Fig. 6f), which verifies the generation of O_2_ during the relithiation. The following equations summarize the redox reactions that likely occurred between the solid NCM and the liquid LiOH solution.

Oxidation:

9 $$4{{{OH}}^{-}}_{({aq})}\to {O}_{2(g)}+2{{H}_{2}O}_{(l)}+4{e}^{-}$$

Reduction:

10 $${{Li}}_{x}{NCM}{O}_{2(s)}+\left(1-x\right){{{Li}}^{+}}_{({aq})}+(1-x){e}^{-}\to {{Li}}_{1.0}{NCM}{O}_{2(s)}$$

Overall:

11 $${{Li}}_{x}{NCM}{O}_{2(s)}+(1-x){{LiOH}}_{({aq})}\to {{Li}}_{1.0}{NCM}{O}_{2(s)}+\frac{1-x}{4}{O}_{2(g)}+\frac{1-x}{2}{H}_{2}{O}_{(l)}$$

It should be mentioned that a short annealing step generally follows the full hydrothermal relithiation to further improve the cycling stability of the relithiated materials. With that, one can achieve a stable electrochemical cycling for NCM111, 523, 622, and even 811, equivalent to that of pristine materials (Fig. S30). The enhanced capacity and improved electrochemical kinetics can be mainly attributed to the elimination of H^+^ within the crystal lattice. To explain this phenomenon, HRTEM was conducted to compare the material structure before and after sintering. The relithiated sample before sintering showed clean surface and clear lattice fringe (Fig. S31a). The fast Fourier transform (FFT) in the selected region displayed a rhomboid pattern, which is consistent with the observation of layered structure viewed along [010] direction. After sintering, the lattice became more clearly defined, indicating the crystallization was improved (Fig. S31b). Thus, it is speculated that a small number of protons might be still bonded on the particle surface after aqueous relithiation, resulting in mild structural distortion, which could be resolved by subsequent sintering (Fig. S32). The short sintering step effectively removes such residual defects and completely recover the microstructure of the NCM materials.

## Discussion

In summary, our study comprehensively explored the thermodynamics and kinetics of protonation processes, and their resulted evolution of compositional and structural defects in different aqueous conditions. Through advanced characterizations and computational studies, we found that the concentration of H^+^ and Li^+^ in aqueous solution greatly affects this evolution. Specifically, when the solution is neutral and lacks Li^+^, H^+^ can intercalate into the layered structure of the material and leach Li^+^ out of NCM particles. This protonation of NCM can cause poor structural stability and electrochemical performance. Especially with the increase of Ni content in NCM, more severe compositional and structural defects were detected. However, by reducing the proton concentration and increasing the Li^+^ concentration in the aqueous solution, the proton-induced effects on D-NCM can be eliminated due to the increased H^+^ intercalation energy barrier, thereby facilitating lithiation reaction. When NCM materials (NCM111, 523, 622, and 811) with Li deficiencies are treated by concentrated LiOH solution, they can exhibit a consistent layered structure from surface to bulk and delivers much-improved battery performance. The kinetics modeling and experimental results suggest that the restoration of Li deficiencies is dominated by charge transfer between transition metals and OH^-^. This work provides a basis for understanding the underlying mechanisms driving LIBs positive materials regeneration and constitutes a significant step towards the rational design of recycling degraded positive materials.

## Methods

### NCM materials delithiation

Defective NCM111was obtained from Argonne National Laboratory. NCM523, 622, and 811 were purchased from Guangdong Canrd New Energy Technology Co., Ltd. To obtain D-NCM111, 523, 622, and 811 with the same concentration of Li deficiencies of 10%, pristine NCM (P-NCM) was mixed with a solution of K_2_S_2_O_8_ (Sigma Aldrich, 99 %) under magnetic stirring at 50 °C for different time. The material was then washed with water and filtered followed by acetonitrile (Sigma Aldrich, 99 %) washing before drying. This delithiation process set the basis for good materials quality control to compare different resulting materials.

### Aqueous processing

1 g of D-NCM materials were subject to 80 mL of 0 M, 0.1 M, 1 M, and 4 M LiOH solutions, respectively. After mixing under magnetical stirring for 5 minutes, they were then transferred to Teflon-lined autoclave without degassing procedures and maintained at 220 °C for 2 h. The resulting NCM powders were washed thoroughly with deionized water and dried at 120 °C in a vacuum oven overnight. Please note that 0M LiOH refers to pure water here. LiOH was purchased from Sigma Aldrich with a purity of 98%.

### Materials regeneration

To fully regenerate the D-NCM, a short annealing of 850 °C with excess 5% of Li_2_CO_3_ (Sigma Aldrich, 98%) for 4 h in oxygen atmosphere after hydrothermal relithiation with 4M LiOH at 220 °C for 2 h (R-NCM), was conducted. The finally regenerated NCM material was denoted as RS-NCM.

### Quantification of bulk-Li content in NCM

In NCM materials, Li^+^ can exist in the form of bulk-Li and surface-Li. Generally, the surface-Li mainly comprises LiOH and Li_2_CO_3_^59,60^. Given the solubility of these compounds, the accurate ratio of bulk-Li to (Ni+Co+Mn) can be obtained by removing surface-Li via water washing (bulk-Li = total-Li – surface-Li). We applied the acid-base titration method to measure the Li^+^ content in the washed solution and thereby ascertain the surface-Li.

Specifically, specific weights (*W* in g) of delithiated (D-NCM), hydrothermal treated with pure water (W-NCM) and 4 M LiOH solution (4M-NCM) materials were dispersed in a specific volume (*A*_*1*_ in mL) of de-ionized water in a sealed container to suppress further contamination, then stirred for a fixed amount of time. The resulting suspension from (*A*_*1*_) is quickly filtered through a vacuum filtration system. To minimize error, a specific volume (*A*_*2*_ in mL) of the filtered analyte solution is titrated against 0.01 M HCl (Sigma Aldrich, 37 %) of a specific concentration (C_*HCl*_ in M). The titration proceeded until two equivalence points appear, then the input volume of HCl solution corresponding to each equivalence point (*V*_*1*_ and *V*_*2*_ in the order of appearance, both in mL) is obtained. These titration curves are illustrated in Fig. S2. The content of surface Li compounds is calculated using the formula provided below:

12 $${LiOH}\left({wt}.\%\right)=\left[\frac{\left(2{V}_{1}-{V}_{2}\right)\times {C}_{{HCl}}\times {M}_{{LiOH}}}{100\times W}\right]\times \left(\frac{{A}_{1}}{{A}_{2}}\right)\times 100({wt}.\%)$$

13 $${{Li}}_{2}C{O}_{3}\left({wt}.\%\right)=\left[\frac{\left({V}_{2}-{V}_{1}\right)\times {C}_{{HCl}}\times {M}_{{{Li}}_{2}{{CO}}_{3}}}{100\times W}\right]\times \left(\frac{{A}_{1}}{{A}_{2}}\right)\times 100\left({wt}.\%\right)$$

Following the removal of surface-Li, the ratio of bulk-Li to (Ni+Co+Mn) can be obtained.

### Materials characterization

The Li content of NCM materials was confirmed by inductively coupled plasma mass spectrometry (ICP-MS, Thermo Fisher Scientific iCAP RQ). The morphology was examined by scanning electron microscope (SEM, FEI XL30). The crystal structure was characterized by X-ray powder diffraction (XRD, Bruker D2 Phaser) with using Cu-Kα radiation. The surface composition was characterized using X-ray photoelectron spectroscopy (XPS) measurements. XPS data were acquired using PHI 5000 VersaProbe II system (Physical Electronics). The thermal stability was determined by thermogravimetric analysis (TGA) with a TA Instrument analyzer (Q 500) using a heating rate of 10 °C/min from room temperature (~25 °C) to 600 °C in dry air. The granular composition distribution was probed by high-angle annular dark-field scanning transmission electron microscopic (HAADF-STEM) combined with electron energy loss spectroscopy (EELS), which was carried out with Thermofisher Talos F200X G2 S/TEM. The intensity of each spectrum was normalized to the highest intensity peak. The gas concentration was measured by gas chromatography (GC, Tracera GC-2010 Plus) equipped with a Molecular Sieve 5Å capillary column and a barrier ionization discharge (BID) detector. In situ NAP-XPS measurement was performed on a SPECS NAP-XPS instrument. The photon source is the monochromatic X-ray source of Al Kα (1486.6 eV). In the OEMS measurement, the dimensions of working electrode (W- and 4M-NCM111), counter electrode and separator were the same as those used for assembling coin cells. 80 μL of electrolyte was used for each cell. Ar was used as the carrier gas. The cell was sealed and connected via the crimped capillary leak toward the MS. Before charging, the cell was rested for 11 h under open circuit conditions.

### Electrochemical characterization

The electrochemical performance was evaluated by fabricating coin cells. The positive electrode was prepared by casting the slurry composed of NCM powder, conductive agent (Super P65) and polyvinylidene fluoride (PVDF) with a mass ratio of 8:1:1 in N-methyl-2-pyrrolidone (NMP) solvent on an Al foil. After drying at 120 °C for 12 h in a vacuum oven, it was punched into circle electrodes with a diameter of 12 mm and calendared. The mass loading of active material was ~3 mg/cm^2^. Type-2032 coin cells were then made inside that glovebox with the prepared positive electrode, Li metal disc (thickness: 1.1 mm) as the anode, LP 40 (1M LiPF_6_ in ethylene carbonate/diethyl carbonate=50:50 (v/v)) as the electrolyte, and trilayer membrane (Celgard 2320, diameter: 13 mm) as the separator. ~70 μL of electrolyte was used for each coin cell. The electrochemical test was performed at room temperature (~25 °C) using Neware cyclers with activation for 4 cycles at a rate of C/10 followed by 50 cycles at a rate of C/3, where the C-rate is defined as full theoretical capacity in one hour. 1C corresponds150, 170, 180, 200 mA/g for NCM111, 523, 622 and 811, respectively. The galvanostatic intermittent titration technique (GITT) was applied to examine the diffusion coefficient of NCM materials at a C/10 rate for current pulses of 10 mins followed by resting periods of 30 min. The cyclic voltammetry (CV) curve of NCM materials in aqueous electrolytes was recorded with a 3-electrodes system. The working electrode was made by coating the slurry prepared as earl mentioned on Ni foam before drying overnight at 120 °C in a vacuum oven. The mass loading is ~10 mg/cm^2^. The volume of electrolyte for measurement is ~80 mL. The reference and counter electrodes were Ag/AgCl and graphite plate, respectively.

### DFT calculations

All density functional theory (DFT) calculations were performed using the Vienna Ab initio simulation (VASP) package with the projected-augmented wave method. The strongly constrained and appropriately normed (SCAN) functional^61^ was used for the description of exchange-correlation interactions. The Hubbard U parameters of 2.43, 2.86 and 2.93 were applied for Ni, Co, and Mn, respectively^62^. The energies and forces were converged to within 10^–5^ eV and 0.02 eV·Å^−1^, respectively, in all structural optimizations and total energy calculations. A *k*-point density of at least 1000/(number of atoms in the unit cell) and an energy cutoff of 520 eV were used. All crystal structure manipulations and data analysis were carried out using the *Pymatgen* software package^63^. We also benchmarked the Perdew-Burke-Ernzerhof with Hubbard U parameters (PBE + U)^64^ and SCAN with van der Waals (vdW) functional and Hubbard U parameters (SCAN + rvv10+U)^65^ in predicting the ground spin state and lattice parameters for Ni, Co and Mn hydroxides. (See Table S1).

### Initial structures and chemical space

All symmetrically distinct Ni/Co/Mn and Li/vacancy/H orderings in O3 NCM structures were represented by a set of special quasi-random structures (SQS)^66^ generated by ATAT toolkit^67^. The Ni, Co and Mn atoms were distributed randomly among 3b Wyckoff sites, while Li, H atoms and vacancies were distributed randomly among the 3a Wyckoff sites. The SQSs were generated using 3√3 × 3√3 × 1 supercells with chemical formula of Li_27-*x*_H_*y*_•_*x*_Ni_9_Co_9_Mn_9_O_54_ (0 ≤ x+y ≤ 27, •: vacancy) and Li_27-*x*-*y*_H_y_•_x_Ni_21_Co_3_Mn_3_O_54_ (0 ≤ *x*+*y* ≤ 27, •: vacancy) for NCM111 and NCM811 materials, respectively.

The materials considered here are compounds with the chemical formula of Li_1-*x*_H_*y*_(NiCoMn)_1/3_O_2_ and Li_1-*x*_H_*y*_(Ni_7/9_Co_1/9_Mn_1/9_)O_2_(0 ≤*x*, *y* ≤ 1). The partially lithiated/protonated NCM structures were studied at composition of $x=3/{\mathrm{9,4}}/{\mathrm{9,5}}/{\mathrm{9,6}}/{\mathrm{9,7}}/{\mathrm{9,8}}/9,y=1-$ x; This selection is based on the experimental (de)-intercalation and protonation ranges conducted in this work. Notably, only the reducing protons (Coleman’s proton)^68^ are considered, as no transition metal vacancies have been observed experimentally. For each chemical composition, we generated a minimum of 30 symmetrically unique SQSs and subjected them to structure relaxation. We then selected the structure with the lowest energy from each composition to calculate the phase diagram, voltage profiles, lithium-ion migration barriers, and solid-gas reactions at the NCM surface. The lattice parameters for each composition were determined by considering the structures with energies within 10 meV/atom of the lowest energy configuration.

The calculated voltage profiles of (de)-lithiation in NCM111 and NCM811 are shown in Fig. S7. The computed average voltage is within 0.5 V of the experimental voltage curves. It should be noted that exact reproduction of the experimental voltage curve (measured at room temperature, ~25 °C) is not possible with DFT calculations at 0K. These results suggest that the computational methods utilized in this study are reliable in providing accurate descriptions of the NCM materials.

### Construction of grand potential phase diagram

We constructed the grand potential phase diagram of Li-Ni-Co-Mn-O-H chemical space, which maps the equilibrium phases as functions of pH and the chemical potential of Li^+^. To create this phase diagram, we integrated all calculated NCM structures and other compounds within the Li-Ni-Co-Mn-O-H chemical space from the Materials Project^69^, as well as the related aqueous ions from the NIST-JANAF database^70^. The construction of the grand potential phase diagram follows the methodology developed by Persson^71^ and Sun et al. ^72^. The stable domains on phase diagram are determined based on the knowledge of all possible equilibrium redox reactions in the chemical composition of interest. The grand potential (*Ψ*) of a material in an aqueous lithium solution is determined by performing a Legendre transformation of the Gibbs free energy with respect to the oxygen chemical potential, ${\mu }_{{{\rm{O}}}}$; the hydrogen chemical potential, ${\mu }_{{H}^{+}}$; and redox potential, *E*, as well as the chemical potential of Li^+^,${\mu }_{{{{\rm{Li}}}}^{+}}$;

14 $${\varPsi }={\mu }_{{{\rm{bulk}}}}-{\mu }_{{{{\rm{Li}}}}^{+}}{N}_{{{\rm{Li}}}}-E{{\rm{Q}}}-{{\mu }_{{{{\rm{H}}}}^{+}}{N}_{{{\rm{H}}}}-\mu }_{{{\rm{O}}}}{N}_{{{\rm{O}}}}$$

In an aqueous system, ${\mu }_{{{{\rm{H}}}}^{+}}$ and ${\mu }_{{{\rm{O}}}}$ are constrained by the water-oxygen equilibrium, which yields

15 $${\mu }_{{{\rm{O}}}}={\mu }_{{{{\rm{H}}}}_{2}{{\rm{O}}}}-2{\mu }_{{{{\rm{H}}}}^{+}}+2E$$

As the number of transition metal atoms (${N}_{{{\rm{M}}}}$) are conserved in the phase transformations between transition metal oxides with different compositions, the normalized *Ψ* can be obtained:

16 $$\bar{\varPsi }=\frac{1}{{N}_{{{\rm{M}}}}}(({\mu }_{{{\rm{bulk}}}}-{\mu }_{{{{\rm{Li}}}}^{+}}{N}_{{{\rm{Li}}}}-{\mu }_{{{{\rm{H}}}}_{2}{{\rm{O}}}}{N}_{{{\rm{O}}}})+(2{N}_{{{\rm{O}}}}-{N}_{{{\rm{H}}}}){\mu }_{{{{\rm{H}}}}^{+}}-(2{N}_{{{\rm{O}}}}-{N}_{{{\rm{H}}}}+Q)E)$$

All analysis were performed at the zero external potential, i.e., *E* = 0 V vs SHE. ${\mu }_{{{{\rm{H}}}}_{2}{{\rm{O}}}}$ is set to -2.46 eV, corresponding to the experimental formation free energy of water at 298 K^70^. The baseline for ${\mu }_{{{{\rm{Li}}}}^{+}}$ is established using the value for 1 M Li^+^ in water at 298 K, derived from the DFT-calculated energy of the Li metal, plus the experimental formation free energy of the lithium ion in water^10^. Then ${\mu }_{{{{\rm{Li}}}}^{+}}$ can be determined by the equation ${\mu }_{{{{\rm{Li}}}}^{+}}={RT}{\mathrm{ln}}({a}_{{{\rm{Li}}}})$, where *R* is the gas constant, *T* is the temperature and ${a}_{{{\rm{Li}}}}$ is the activity of Li^+^ in the solution. The referenced energy of ${\mu }_{{{{\rm{H}}}}^{+}}$ is determined by ${\mu }_{{{{\rm{H}}}}^{+}}$ + e^−^ = ½ H_2_(g). ${\mu }_{{{{\rm{H}}}}^{+}}$ is transformed into pH using the relationship ${\mu }_{{{{\rm{H}}}}^{+}}=-{RT}{\mathrm{ln}}(10){{\rm{pH}}}$.

### The chemical potential of solid phases

The chemical potential of a solid material under standard conditions is given by,

17 $${\mu }_{{{\rm{bulk}}}}^{0}={H}^{0}-T{S}^{0}={E}_{{{DFT}}}+{E}_{{{ZPE}}}+\delta H-T{S}^{0}$$

where ${E}_{{DFT}}$ is the DFT calculated total energy, ${E}_{{ZPE}}$ is the zero-point energy and *δH* is the integrated heat capacity from 0 to 298 K, and $T{S}^{0}$ is the entropy contribution at the standard conditions. The *E*_*ZPE*_ and *δH* for both solid and gas species were neglected, based on the assumption that these energies contribute similarly across species, hence offsetting each other in reaction energies^73^. While the entropy contribution is generally negligible for most solid species due to its minimal value, the configurational entropy in disordered NCM structures might be significant when compared to the enthalpy differences between competing structures^74^. Therefore, the chemical potential for disordered solid species is calculated by,

18 $${G}_{{{\rm{solid}}}}={E}_{{{\rm{DFT}}}}-T{S}_{{{\rm{config}}}}$$

The configurational entropy (*S*_config_) is determined by considering the arrangements of the Li/H/Vacancy and Ni/Co/Mn in their sublattices according to the following definition:

19 $${S}_{{{\rm{config}}}}=\frac{-R{\sum }_{{{\rm{s}}}}{\sum }_{{{\rm{i}}}}{{{\rm{a}}}}^{{{\rm{s}}}}{X}_{{{\rm{i}}}}^{{{\rm{s}}}}{{\mathrm{ln}}}({X}_{{{\rm{i}}}}^{{{\rm{s}}}})}{{\sum }_{{{\rm{s}}}}{{{\rm{a}}}}^{{{\rm{s}}}}}$$

where ${{{\rm{a}}}}^{{{\rm{s}}}}$ is the number of sites on the s sublattice, and ${X}_{{{\rm{i}}}}^{{{\rm{s}}}}$ is the fraction of element species i randomly distributed on the sublattice.

### Chemical potential of aqueous ion

The chemical potential of aqueous ionic species was derived from experimental data, adjusted for the energy difference between the reference solid phase determined as by DFT calculations and experimental measurements,

20 $${\mu }_{i}^{0}={\mu }_{i\left({aq}\right)}^{0,\exp }+\left[{\Delta {{\rm{g}}}}_{{solid}}^{0,{DFT}}-{\Delta {{\rm{g}}}}_{{solid}}^{0,\exp }\right]$$

### Voltage profiles

The intercalation potential of Li into NCM cathodes *versus* Li/Li^+^ was calculated by^75^

21 $$V=-\frac{{E}_{{DFT}}\left({{Li}}_{n}M\right)-{E}_{{DFT}}\left({{Li}}_{n-x}M\right)-x{E}_{{DFT}}\left({Li}\right)}{{xe}}$$

where *E*_DFT_ denotes the DFT calculated total energy and *e* is the electron charge.

### NEB calculations

The lithium vacancy migration barriers were calculated using the climbing image nudged elastic band (CI-NEB) method^71^. The SCAN functional without U value was adopted to avoid possible mixing of the diffusion barrier with a charge-transfer barrier^61^. The energies and forces were converged to within 5 × 10^–5^ eV and 0.05 eV Ǻ^–1^, respectively.

## Supplementary information

Supplementary Information

Description of Additional Supplementary Files

Supplementary Dataset 1

Supplementary Dataset 2

Transparent Peer Review file

## Source data

Source Data

## Data Availability

The source data generated in this study are provided in the Source Data file. Source data are provided with this paper.
