# Supplementary material for: Proton-exchange induced reactivity in layered oxides for lithium-ion batteries
Source: Nat Commun. 2024 Nov 13;15:9842. doi: 10.1038/s41467-024-53731-2 (PMC11560953; doi:10.1038/s41467-024-53731-2)
Supplement: Supplementary file 1 — Supplementary Information [file 41467_2024_53731_MOESM1_ESM.pdf]

Supplementary Information

Proton-Exchange Induced Reactivity in Layered Oxide Cathodes for Lithium-ion Batteries

Panpan Xu,[1,2,†] Xingyu Guo,[3,4†] Binglei Jiao,[2] Jinxing Chen,[5] Minghao Zhang,[1] Haodong Liu,[1] Xiaolu Yu,[1] Maura Appleberry,[1] Zhenzhen Yang,[6] Hongpeng Gao,[3] Fan Yang,[7] Yanbin Shen,[2] Jing Gu,[8] Christopher Brooks,[9] Ying Shirley Meng,[1,3,8] Shyue Ping Ong,[1,3,8*] Zheng Chen[1,3,8*]

[1]Aiiso Yufeng Li Family Department of Chemical and Nano Engineering, University of California, San Diego, La Jolla, CA 92093, USA

[2]Suzhou Institute of Nano-Tech and Nano-Bionics, Chinese Academy of Sciences, Suzhou, 215123, P. R. China

[3]Program of Materials Science and Engineering, University of California, San Diego, La Jolla, CA 92093, USA

[4]Key Laboratory for Computational Physical Sciences (MOE), Institute of Computational Physics, Department of Physics, Fudan University, Shanghai 200433, China

[5]Institute of Functional Nano & Soft Materials (FUNSOM), Jiangsu Key Laboratory for Carbon-Based Functional Materials & Devices, Soochow University, Suzhou, 215123, Jiangsu, P. R. China

[6]Chemical Sciences and Engineering Division, Argonne National Laboratory, 9700 South Cass Avenue, Lemont, IL 60439, USA

[7]Department of Chemistry and Biochemistry, San Diego State University, San Diego, CA, USA

[8]Sustainable Power and Energy Center, University of California, San Diego, La Jolla, CA 92093, USA

[9]Honda Development and Manufacturing of America, North American Auto Development Center, 21001 OH-739, Raymond, OH 43067-9705, USA

[†]P. X. and X. G. contributed equally to this work.

Corresponding to zhc199@ucsd.edu (Z. C.), ongsp@ucsd.edu (S.O.)

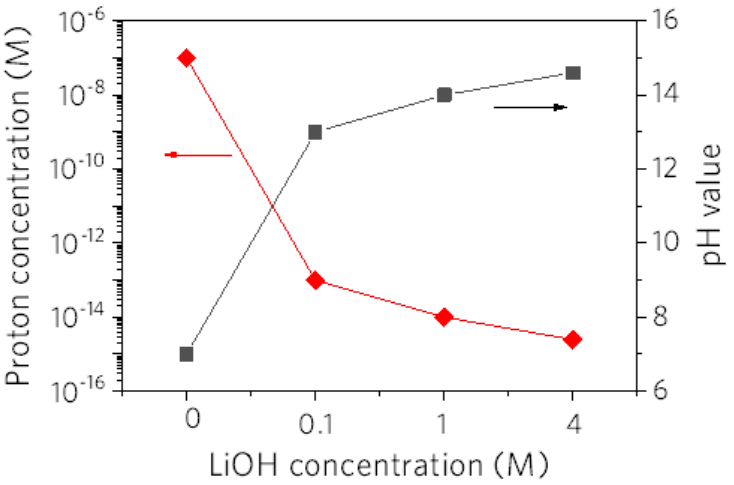

Figure S1. The proton concentrations (red point) and pH values (black point) of 0M, 0.1M, 1M and 4M LiOH solution. Source data are provided as a Source Data file.

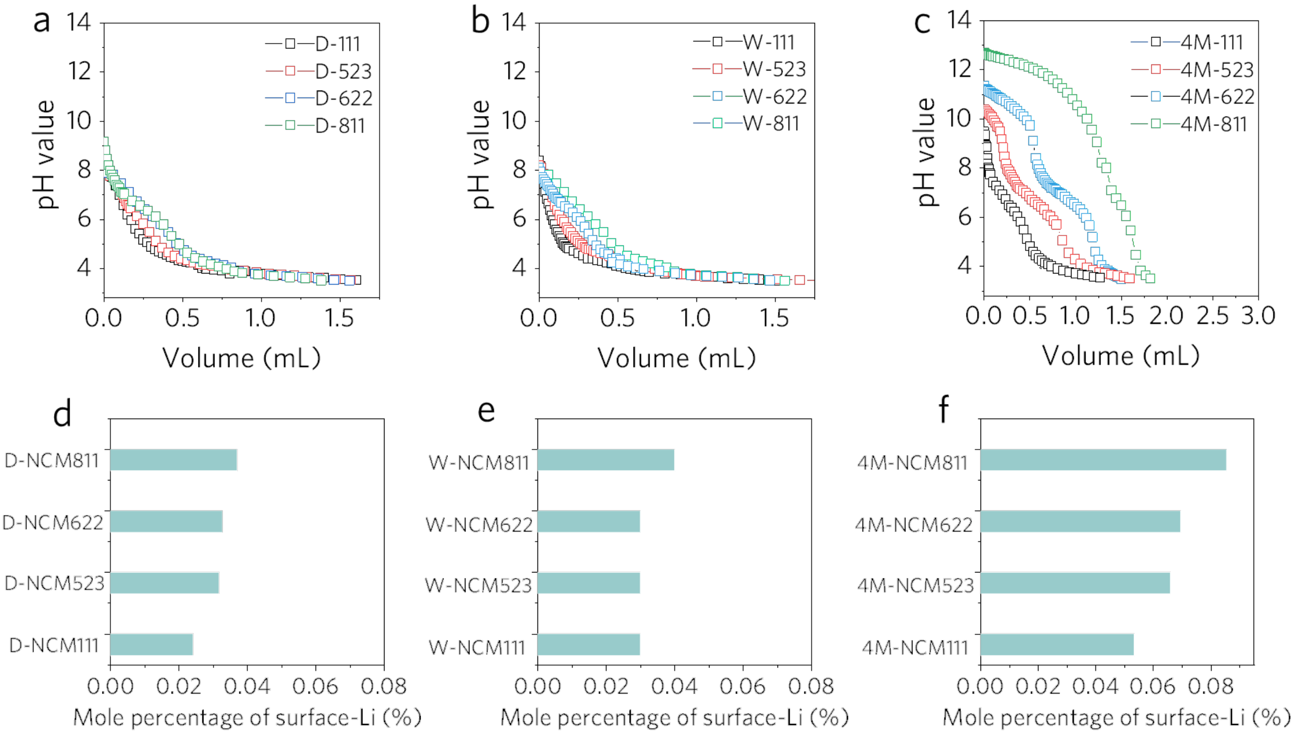

Figure S2. The titration curve of D-NCM (a), W-NCM (b) and 4M-NCM (c); The calculated mole ratio of surface-Li of D-NCM (d), W-NCM (e) and 4M-NCM (f). Source data are provided as a Source Data file.

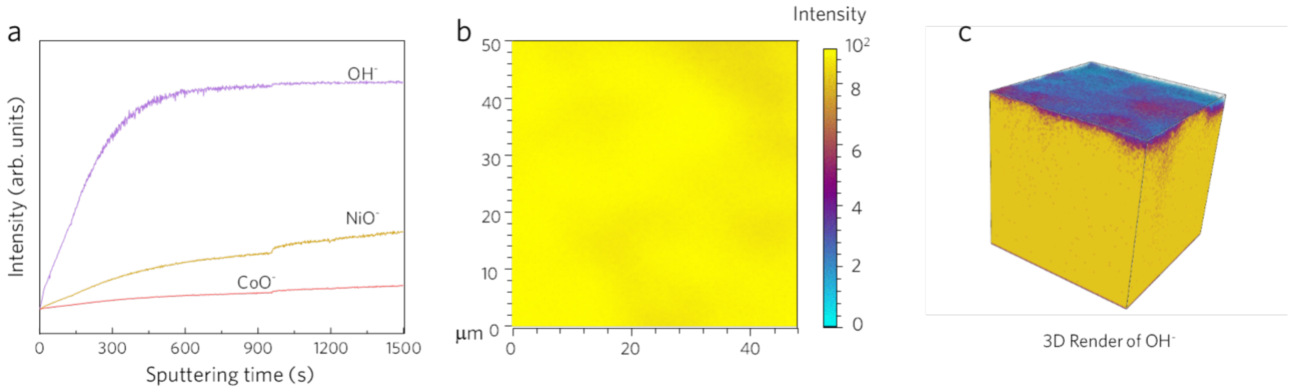

Figure S3. TOF-SIMS spectra of OH^-, NiO^-, CoO^- secondary ion fragments over 1500 s Cs^+ sputtering along the depth profile of the sample after scavenging processes (a); TOF-SIMS chemical mapping of the NCM after the scavenging process, showing the distribution of OH^- secondary ions (b); 3D rendering TOF-SIMS fragments of OH^- (c). Source data are provided as a Source Data file.

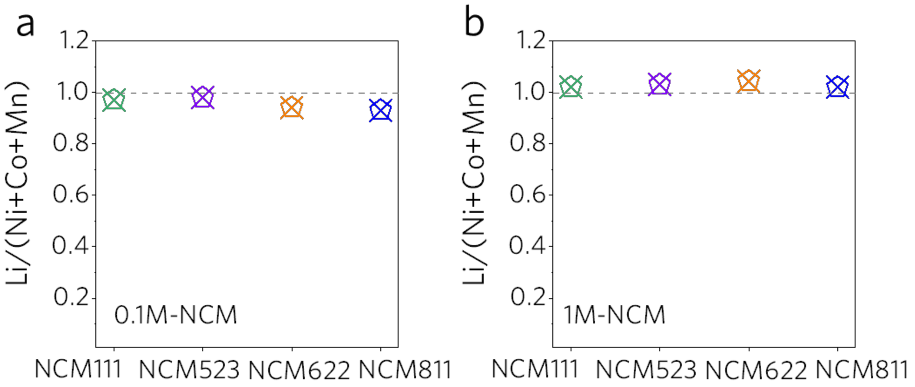

Figure S4. Li^+ concentrations in NCM111, 523, 622 and 811 after treatment with 0.1M (a) and 1M LiOH (b) solutions. Source data are provided as a Source Data file.

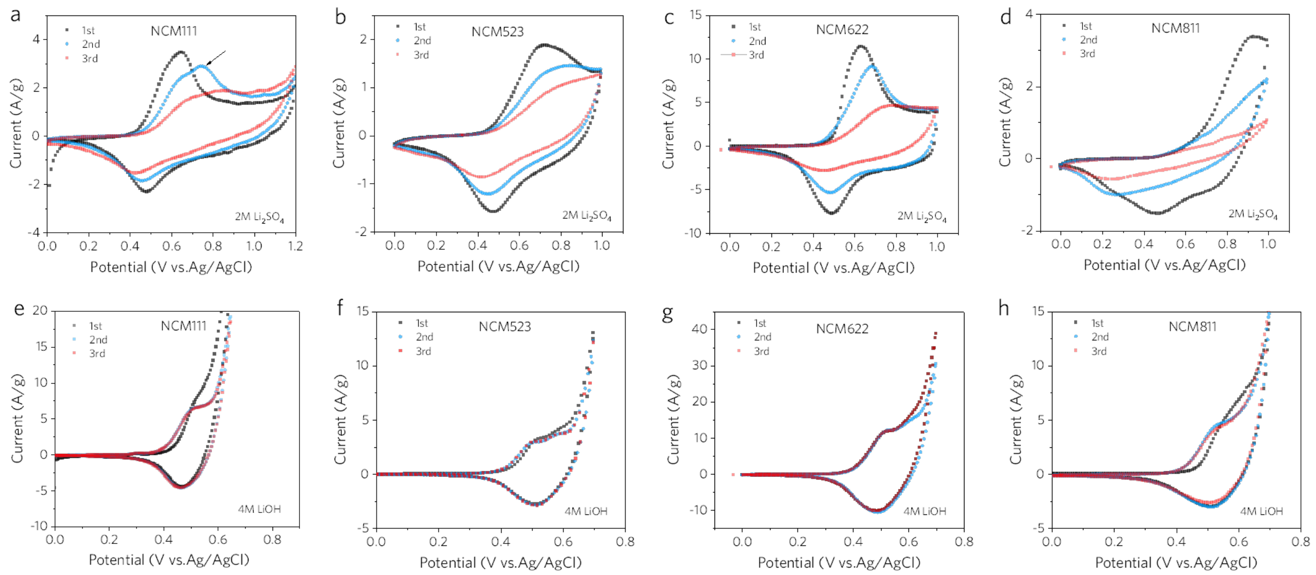

Figure S5. Cyclic voltammograms of different electrodes in different Li-containing electrolytes. The mass loadings of NCM111, 523, 622 and 811 electrodes are 10.54, 11.23, 9.86, and 10.28 mg/cm^2, respectively. Ag/AgCl electrode and graphite plate electrode are used as the reference and counter electrodes, respectively.

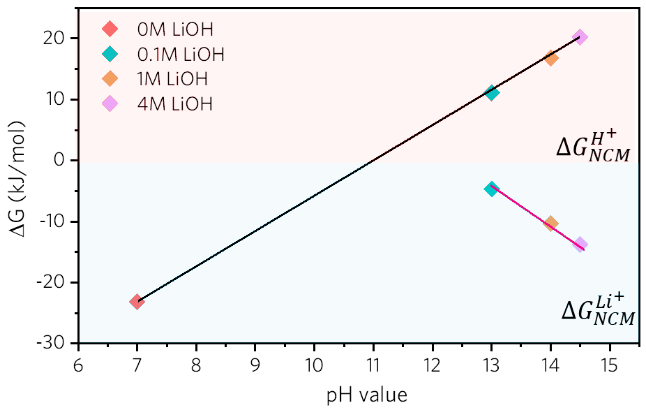

Figure S6. Calculated *Gibbs* free energy for H^+ and Li^+ intercalation in solutions of 0M, 0.1M, 1M and 4M LiOH. Source data are provided as a Source Data file.

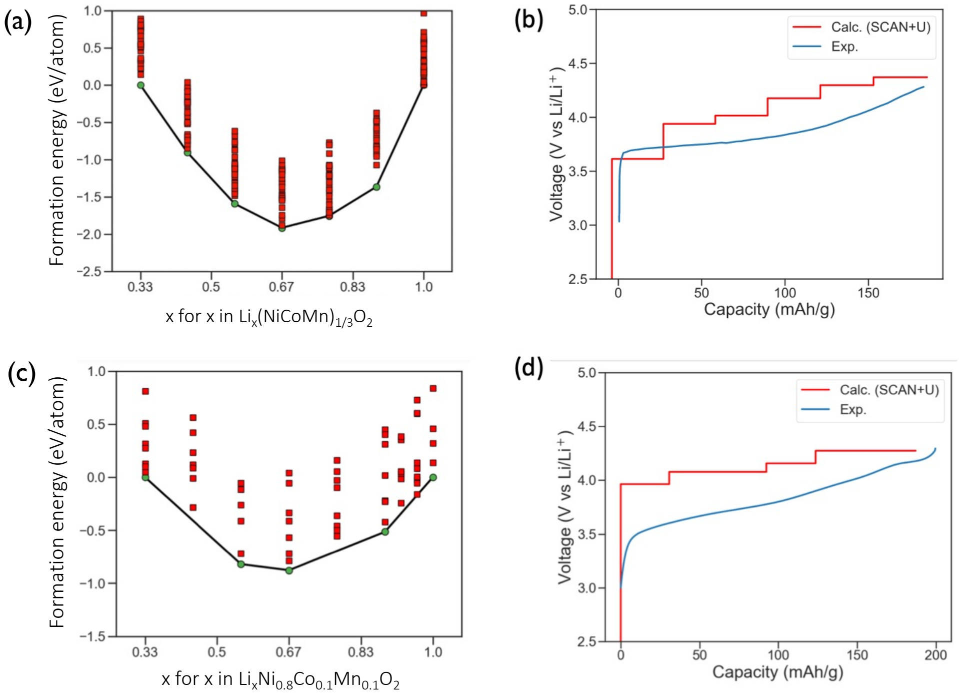

Figure S7. Calculated pseudo-binary phase diagrams and derived voltage profiles for NCM111 and NCM811 cathodes. DFT pseudo-binary phase diagram (a) and voltage profile for NMC111 (b); DFT pseudo-binary phase diagram (c) and voltage profile for NMC811 (d). The voltage range is 3-4.3 V. Source data are provided as a Source Data file.

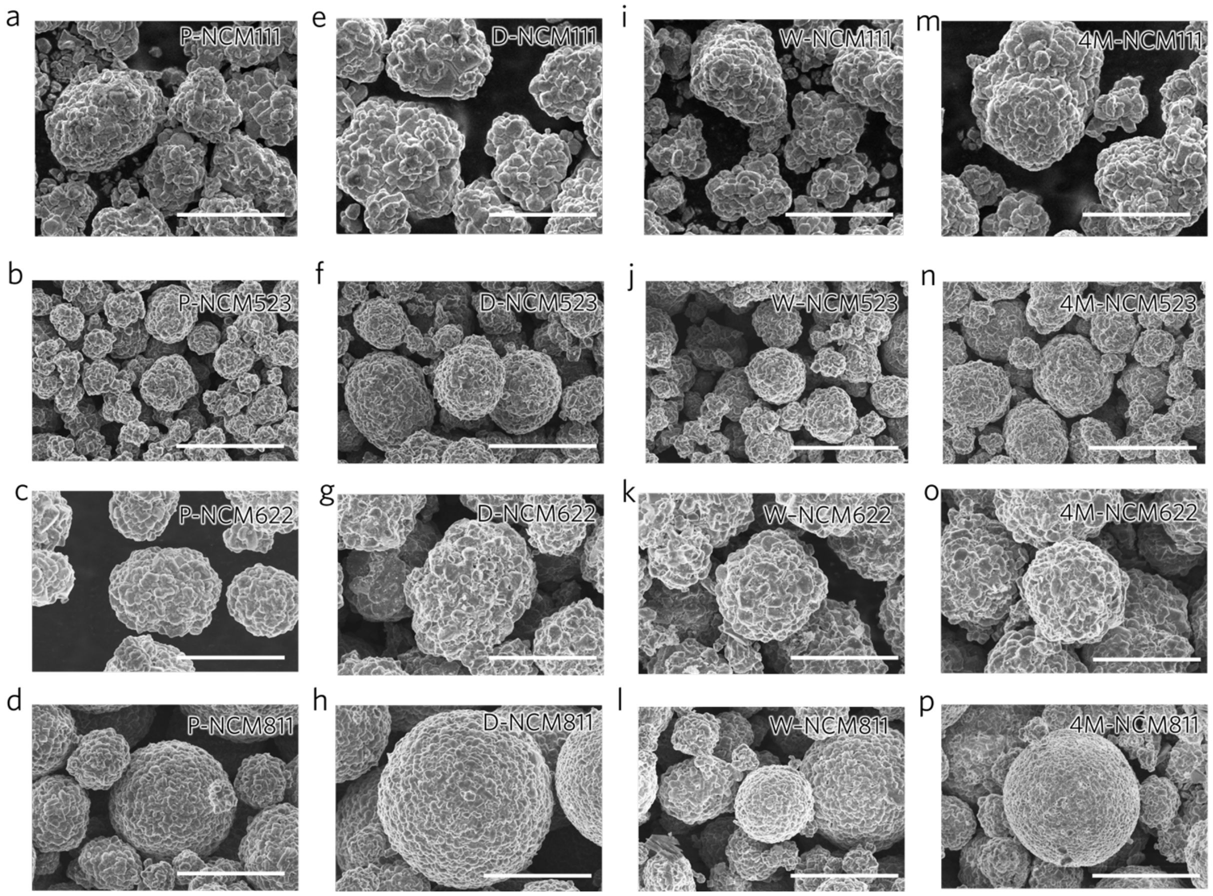

Figure S8. SEM images of T-NCM (a-d), D-NCM (e-h), W-NCM (i-l) and 4M-NCM (m-p).

The scale bar is 10 μm. All the P-NCM materials exhibited a spherical secondary particle shape with a diameter ranging from 3 to 10 μm, comprising small particles with an average diameter of approximately 1.5 μm (a-d). Notably, the morphology of the NCM following chemical delithiation remained largely unchanged compared to the pristine P-NCM (e-h). Furthermore, subsequent treatment with pure water and the 4M LiOH solution did not significantly influence the sample morphology (i-p).

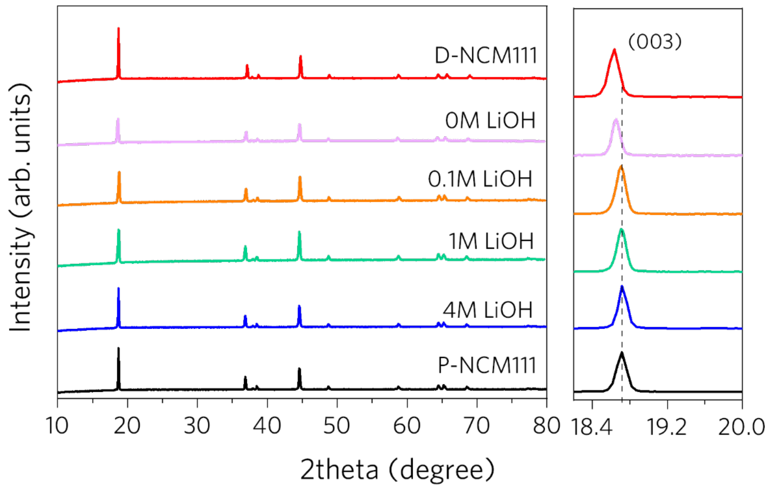

Figure S9. XRD patterns of NCM111 treated with pure water, 0.1M, 1M, and 4M LiOH solutions, as well as the pristine P-NCM111and D-NCM111. Source data are provided as a Source Data file.

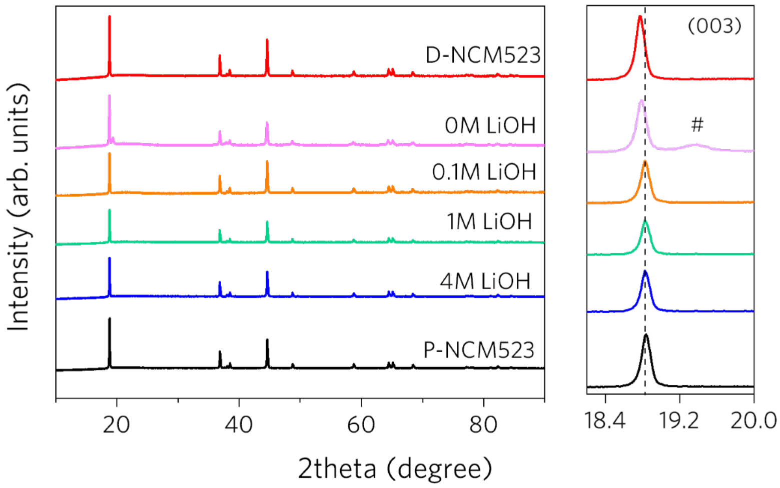

Figure S10. XRD patterns of NCM523 treated with pure water, 0.1M, 1M, and 4M LiOH solutions, as well as the pristine P-NCM523 and D-NCM523. Source data are provided as a Source Data file.

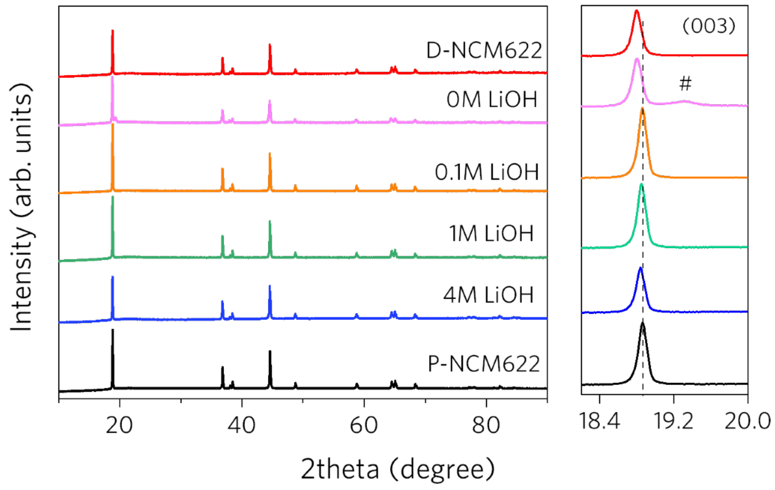

Figure S11. XRD patterns of NCM622 treated with pure water, 0.1M, 1M, and 4M LiOH solutions, as well as the pristine P-NCM622 and D-NCM622. Source data are provided as a Source Data file.

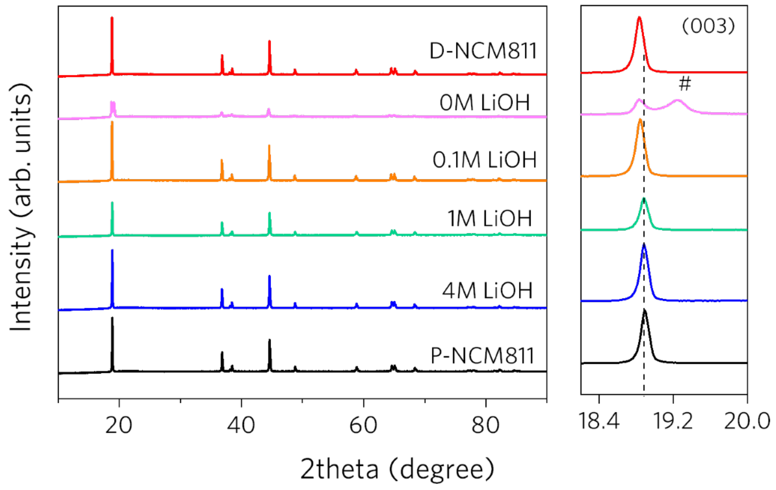

Figure S12. XRD patterns of NCM811 treated with pure water, 0.1M, 1M, and 4M LiOH solutions, as well as the pristine P-NCM811 and D-NCM811. Source data are provided as a Source Data file.

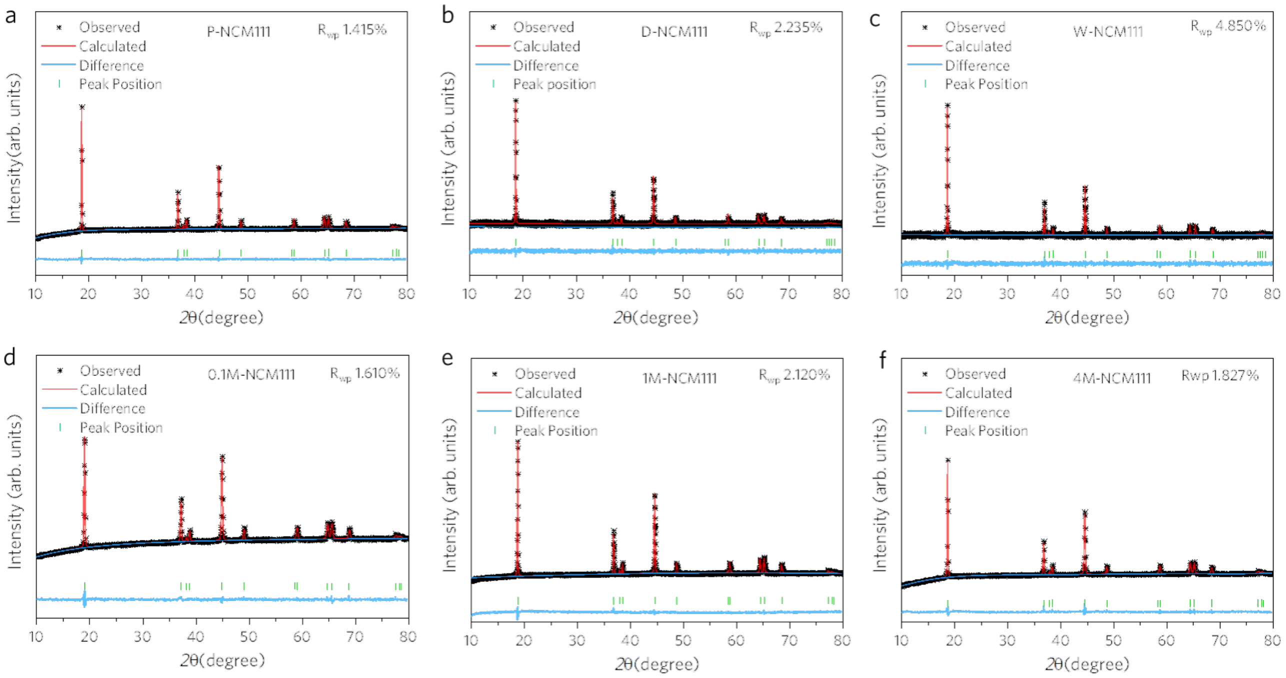

Figure S13. Rietveld refinement of XRD patterns of P-NCM111 (a) and D-NCM111 (b), as well as the resulting NCM111 after treatment with 0M LiOH (c), 0.1M LiOH (d), 1M LiOH (e), and 4M LiOH (f). Source data are provided as a Source Data file.

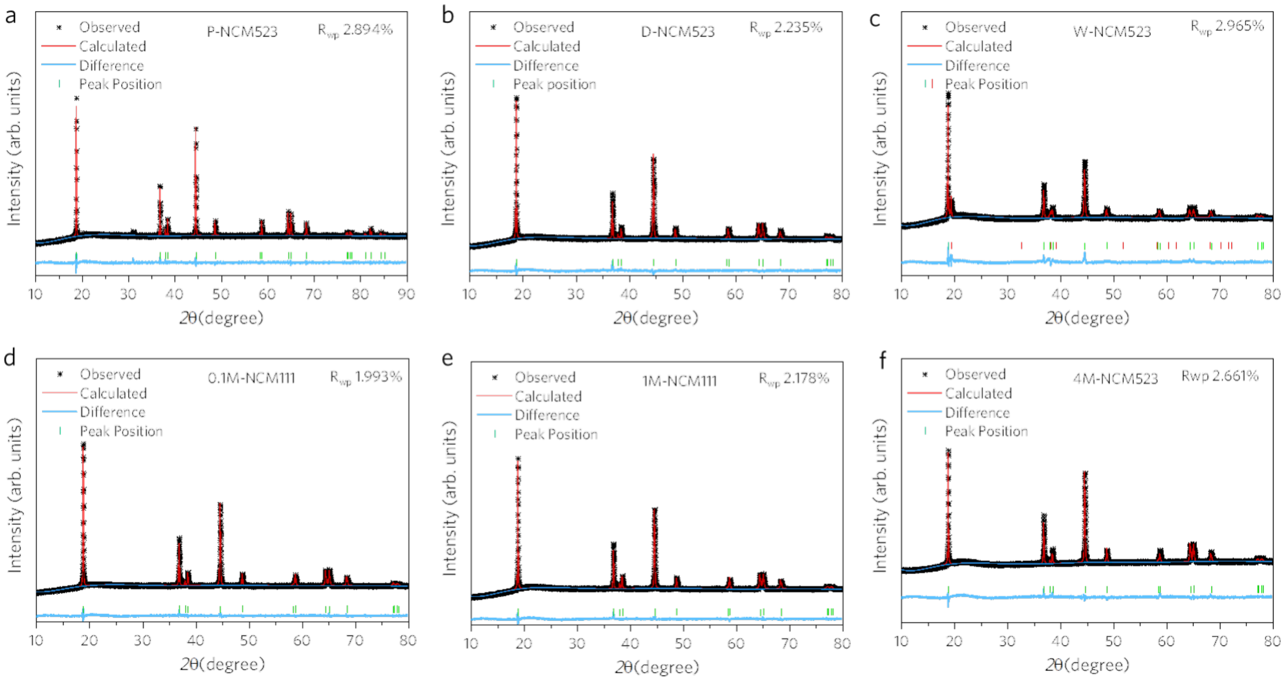

Figure S14. Rietveld refinement of XRD patterns of P-NCM523 (a) and D-NCM111 (b), as well as the resulting materials after treatment with 0M LiOH (c), 0.1M LiOH (d), 1M LiOH (e), and 4M LiOH Source data are provided as a Source Data file.

(f).

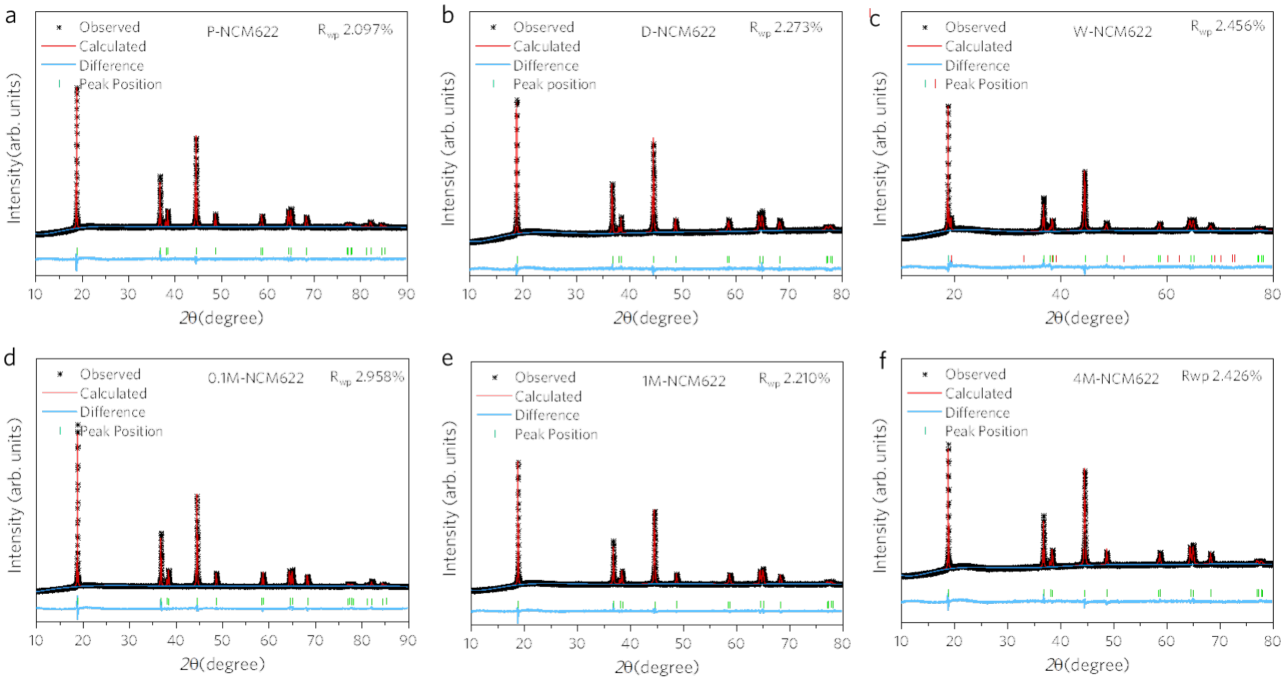

Figure S15. Rietveld refinement of XRD patterns of P-NCM622 (a) and D-NCM111 (b), as well as the resulting materials after treatment with 0M LiOH (c), 0.1M LiOH (d), 1M LiOH (e), and 4M LiOH (f). Source data are provided as a Source Data file.

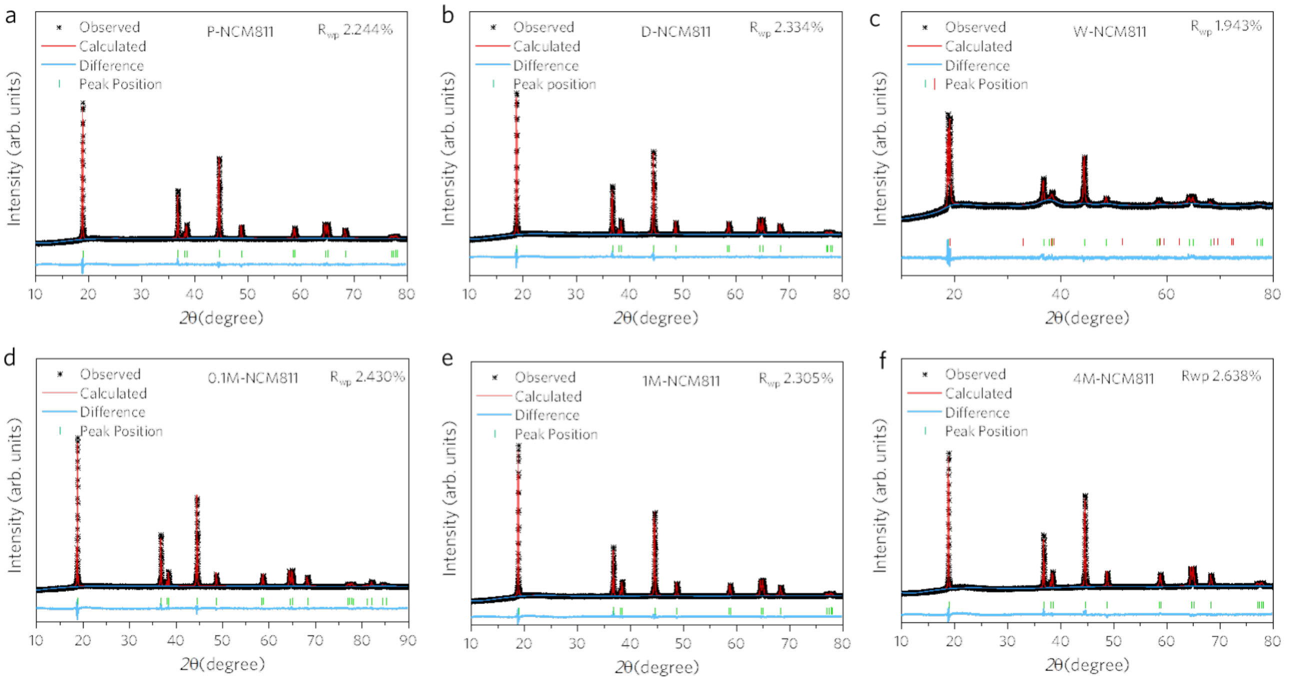

Figure S16. Rietveld refinement of XRD patterns of P-NCM811 (a) and D-NCM111 (b), as well as the resulting materials after treatment with 0M LiOH (c), 0.1M LiOH (d), 1M LiOH (e), and 4M LiOH (f). Source data are provided as a Source Data file.

Table S1 Calculated ground spin state, lattice parameters (error compared with experimental data) of cobalt, nickel and manganese hydroxides with different functionals.

Material	Group space	Ground spin state	Exp.	Functionals		
				PBE + U	SCAN + U	SCAN + rvv10 + U
NCM111	R-3m	Ni^{2+}(LS) Co^{3+}(LS) Mn^{4+}(HS)	a = 2.868 c=14.240	a=2.849 (-0.07%) c=14.39 (1.20%)	a=2.841 (-0.35%) c=14.159 (-0.40%)	a=2.858 (0%) c=14.134 (-0.6%)
NCM811	R-3m	Ni^{2+}(LS) Ni^{3+}(LS) Co^{3+}(LS) Mn^{4+}(HS)	a = 2.871 c = 14.199	a = 2.89 (0.72%) c = 14.35 (1.09%)	a = 2.85 (-0.57%) c = 14.10 (-0.71%)	a = 2.848 (-0.79%) c = 14.095 (-0.73%)
$CoHO_2$	R-3m	Co^{3+}(LS)	a = 2.851 c = 13.15	a=2.849 (-0.07%) c=12.935 (-1.63%)	a=2.841 (-0.35%) c=2.986 (-1.29%)	a=2.836 (-0.53%) c=2.979 (-1.30%)
$Co(HO)_2$	C2/m	Co^{2+}(HS)	a = 3.186 b = 3.186 c = 4.653	a=3.209 (0.91%) b=3.225 (1.42%) c=4.779 (2.71%)	a=3.162 (-0.57%) b=3.162(-0.57%) c=4.593 (-1.29%)	a=3.158 (-0.69%) b=3.157 (-0.72%) c=4.516 (-2.94%)
$MnHO_2$	Pnma	Mn^{3+}(HS)	a = 2.871 b = 4.554 c = 10.667	a=2.933 (2.16%) b=4.607 (1.16%) c=10.925 (2.42%)	a=2.91 (1.36%) b=4.556 (0.04%) c=10.604 (-0.59%)	a=2.905 (1.18%) b=4.541 (-0.29%) c=10.572 (-0.89%)
$MnHO_2$	$P2_1/c$	Mn^{3+}(HS)	a = 5.304 b = 5.277 c = 5.304	a=5.375 (1.34%) b=5.66 (7.26%) c=5.383 (1.49%)	a=5.307 (0.06%) b=5.238 (-0.74%) c=5.308 (0.08%)	a=5.294 (-0.19%) b=5.221 (-0.74%) c=5.308 (0.08%)
$NiHO_2$	C2	Ni^{3+}(LS)	a = 3.1268 c = 9.212	a=3.165 (1.22%) c=9.354 (1.54%)	a=3.113 (-0.44%) c=8.919 (-3.18%)	a=3.113 (-0.44%) c=8.919 (-3.18%)

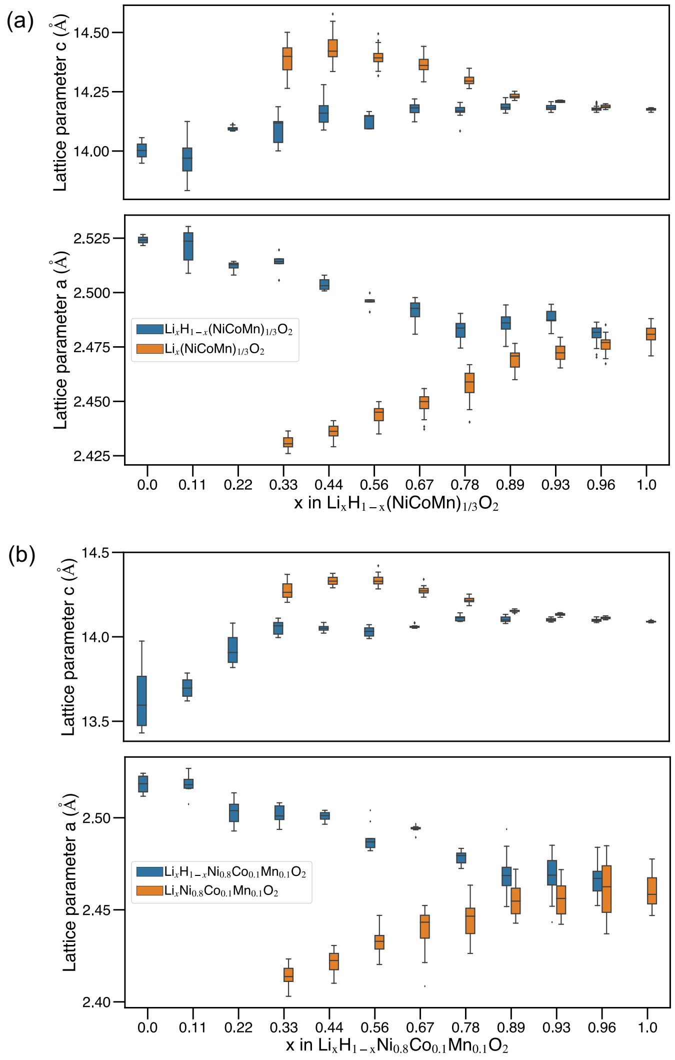

Figure S17. Changes of lattice parameters of lithiated and protonated NCM111 (a) and NCM811 (b) structures. During the de-lithiation process, the *c* lattice parameters of both cathodes first expand and then contract when more than 0.66 Li has been extracted from the cathodes. The expansion of *c*-axis during the de-lithiation is attributed to the increased the repulsion effect between adjacent oxygen planes; while the subsequently shrinkage of *c* axis is likely due to the combined effects of the dissipation of effective charge on oxygen and residual nonlocal dispersion forces from lithium vacancies.[1] In contrast, the c lattice parameters of protonated NCM cathodes slightly decrease as more lithium is deintercalated, while more protons are inserted simultaneously. This is likely because of the formation of hydrogen bonds between protons and oxygen atoms in adjacent layers. In addition, the repulsion effect between adjacent oxygen planes can also be reduced due to the formation of O-H bond between protons and their adjacent oxygen atoms. The whiskers extend to 1.5 times the interquartile range (IQR) from the first and third quartiles (Q1 and Q3). The lattice parameters were calculated using the five lowest-energy configurations for each composition. Source data are provided as a Source Data file.

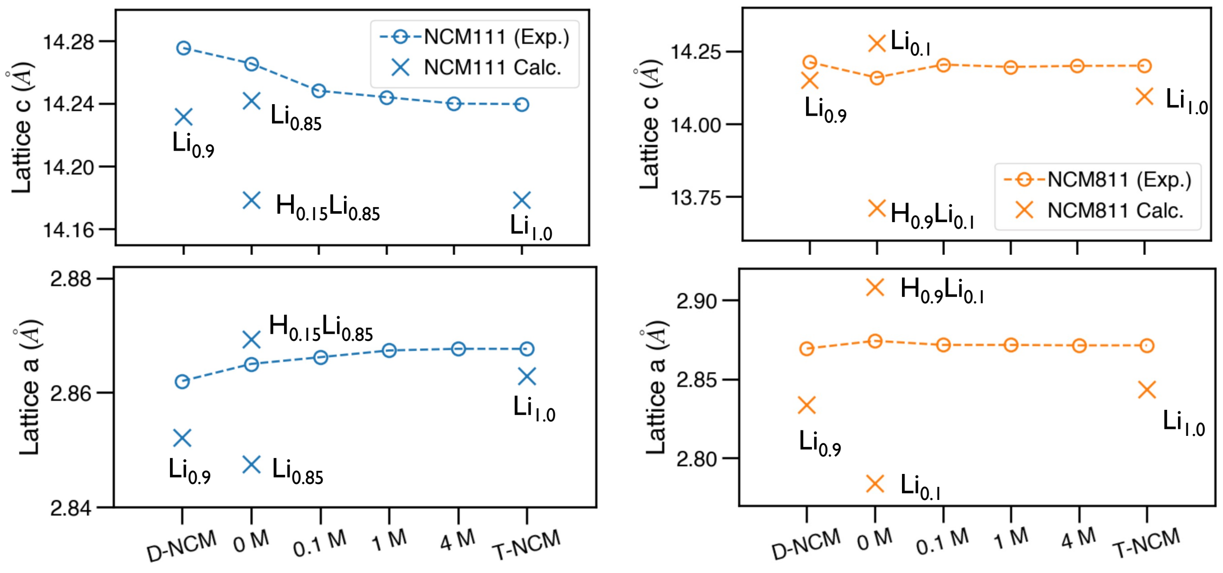

Figure S18. Lattice parameters of NCM111 (a) and NCM811 (b) treated by various LiOH solutions. The calculated lattice parameters of structures with equal lithium content measured by experiment are shown as comparison. Source data are provided as a Source Data file.

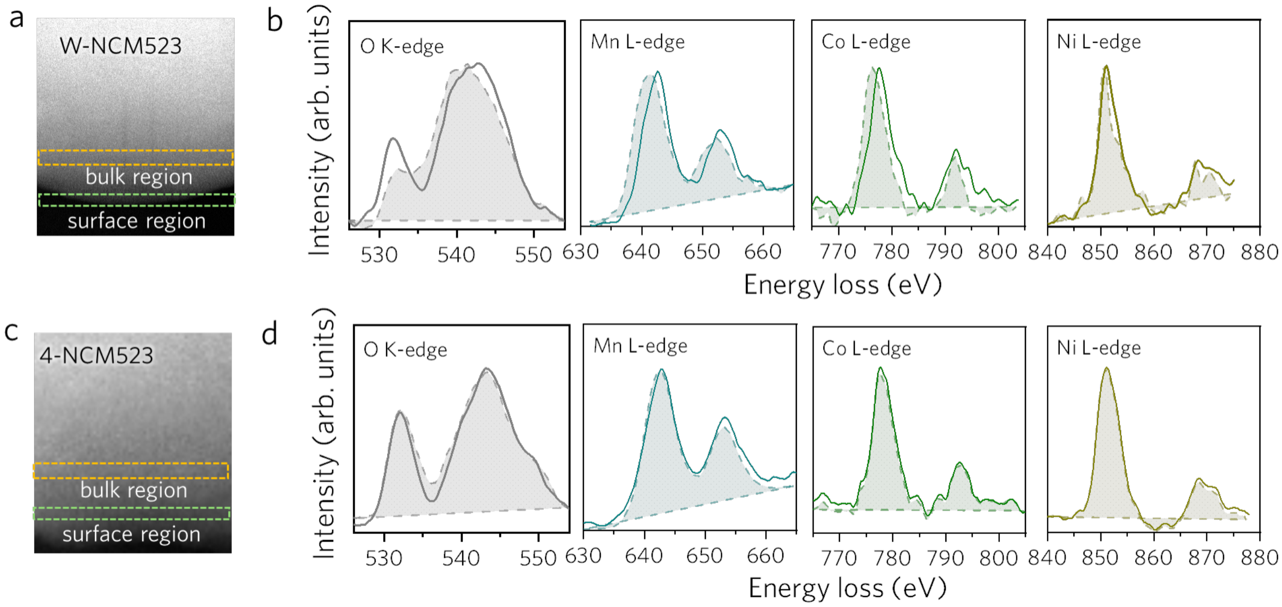

Figure S19. HAADF-STEM images and spatially resolved EELS spectra from the bulk to the surface pure water (a and b) and 4M LiOH solution (c and d) treated NCM523. In figures a and c, the green dashed box highlights the surface region of the materials, while the orange dashed box indicates the bulk region. The EELS spectra obtained from the surface regions are shown as solid lines, whereas spectra from the bulk regions are depicted as dashed lines with shaded regions. Source data are provided as a Source Data file.

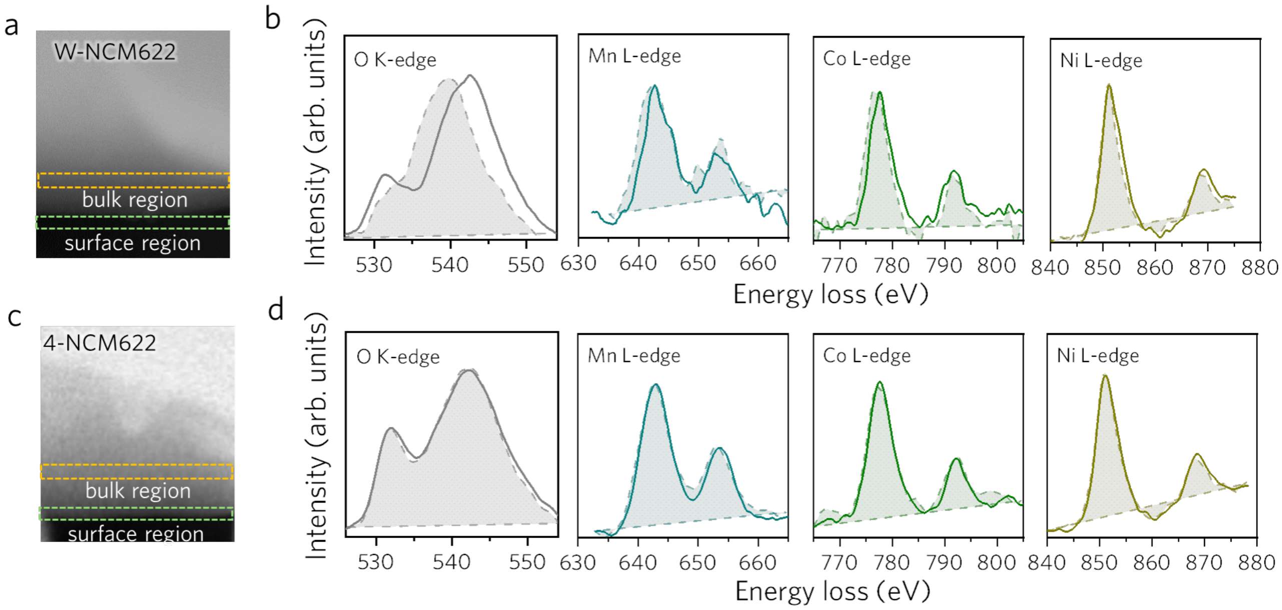

Figure S20. HAADF-STEM images and spatially resolved EELS spectra from the bulk to the surface pure water (a and b) and 4M LiOH solution (c and d) treated NCM622. In figures a-d, the green dashed box highlights the surface region of the materials, while the orange dashed box indicates the bulk region. The EELS spectra obtained from the surface regions are shown as solid lines, whereas spectra from the bulk regions are depicted as dashed lines with shaded regions. Source data are provided as a Source Data file.

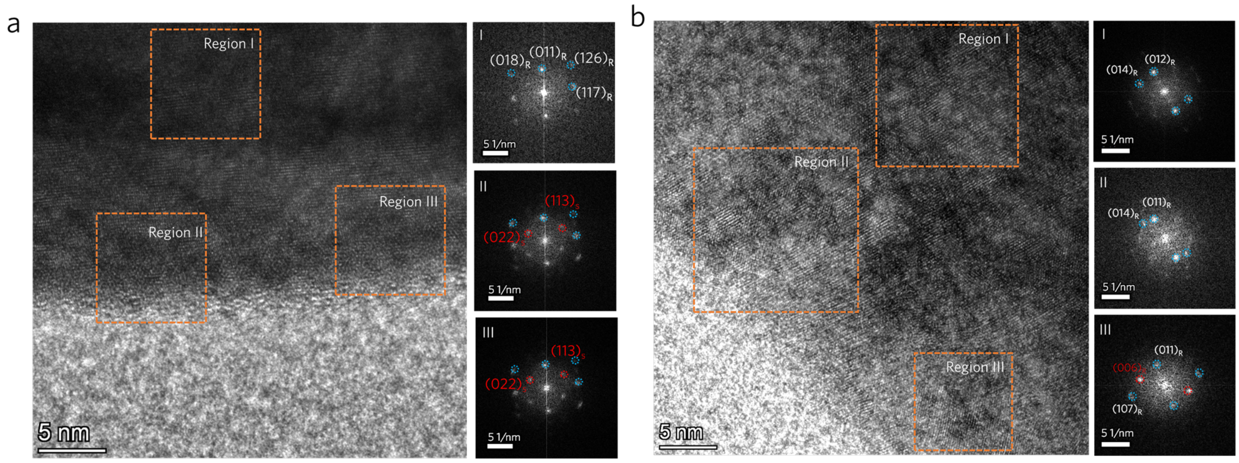

Figure S21. HRTEM and FFT in the selected regions of W-NCM111 (a) and W-NCM111 811 (b). Source data are provided as a Source Data file.

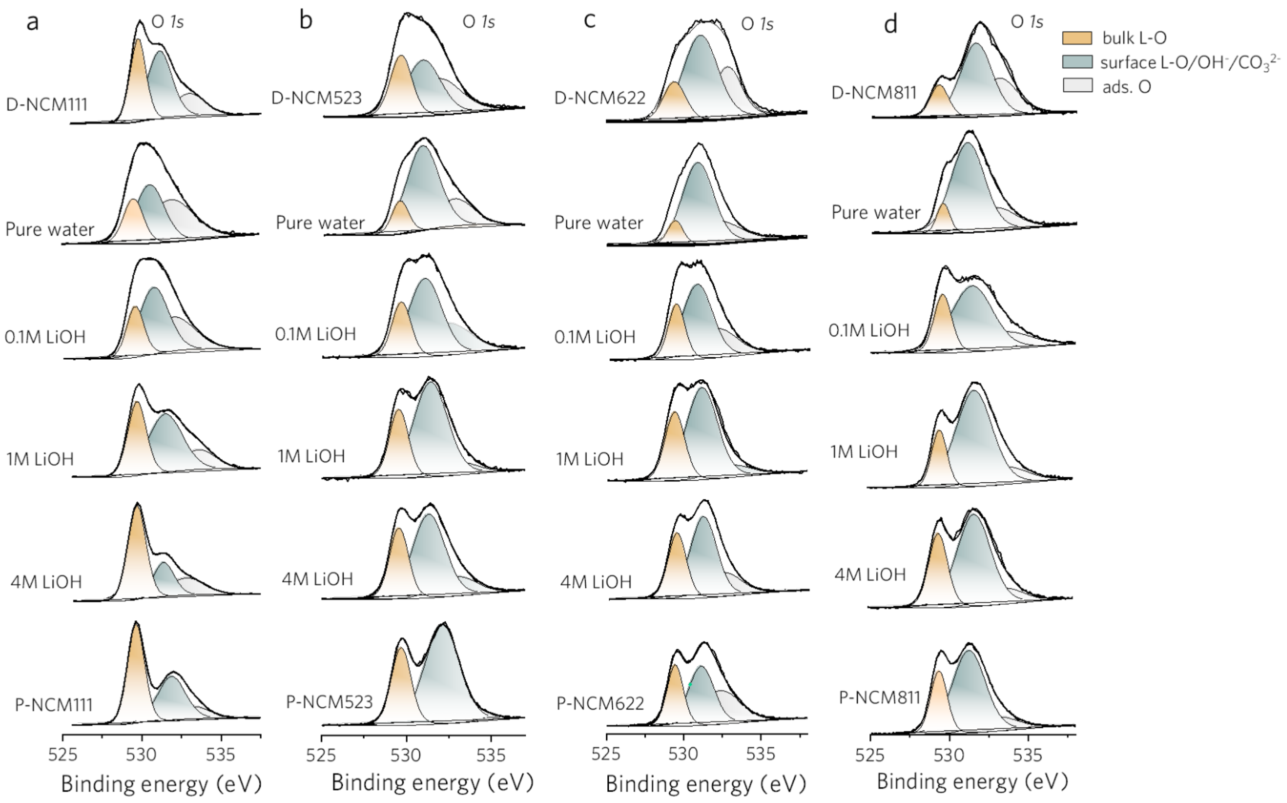

Figure S22. Deconvolution of O 1s XPS spectra of NCM111, 523, 622 and 811 materials treated by pure water, 0.1M, 1M, and 4M LiOH solutions, as well as P-NCM and D-NCM. Source data are provided as a Source Data file.

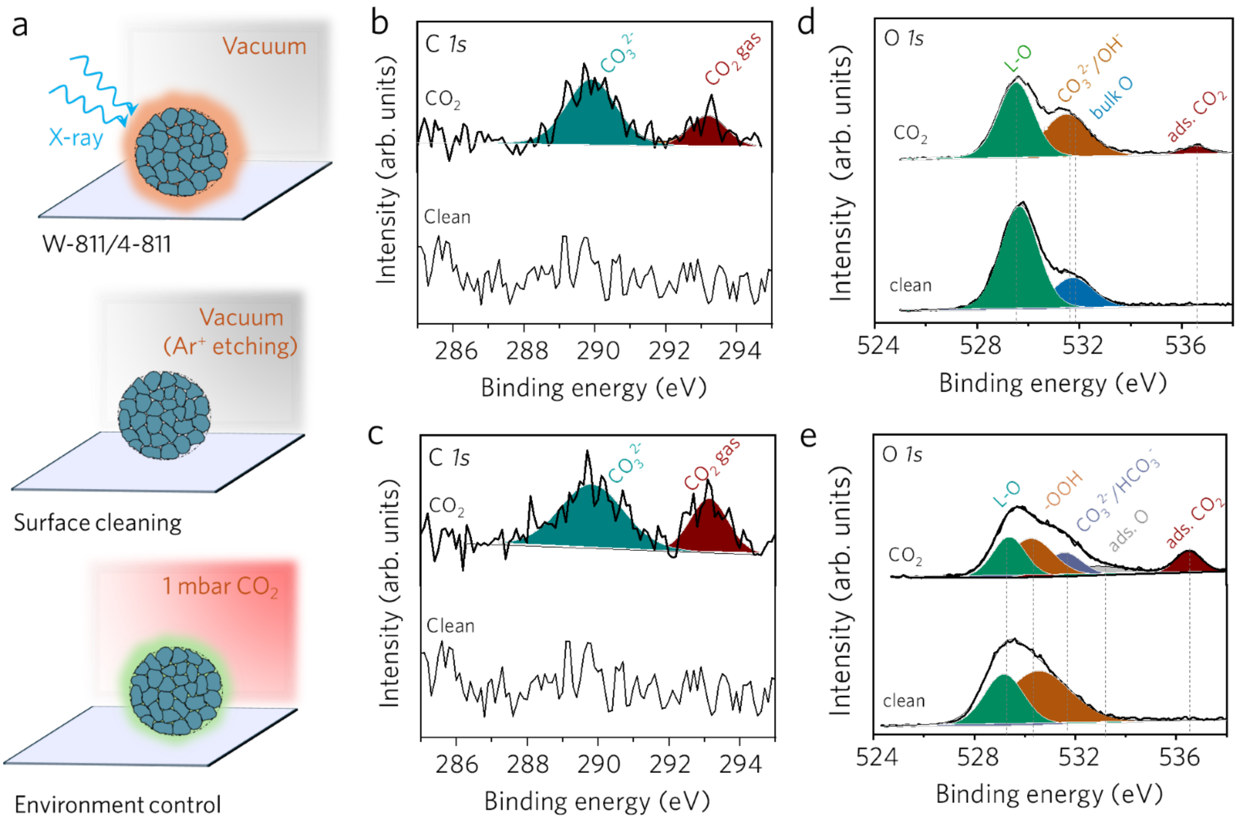

Figure S23. Schematic illustration of in situ NAP-XPS study of interfacial reaction protonated and relithiated NCM811 cathode and CO_2 (a); Deconvolution of the obtained C 1s XPS spectra (b), and O 1s XPS spectra (c) of 4M-NCM811 following exposure to CO_2; Deconvolution of the obtained C 1s XPS spectra (d), and O 1s XPS spectra (e) of protonated NCM811 following exposure to CO_2. Source data are provided as a Source Data file.

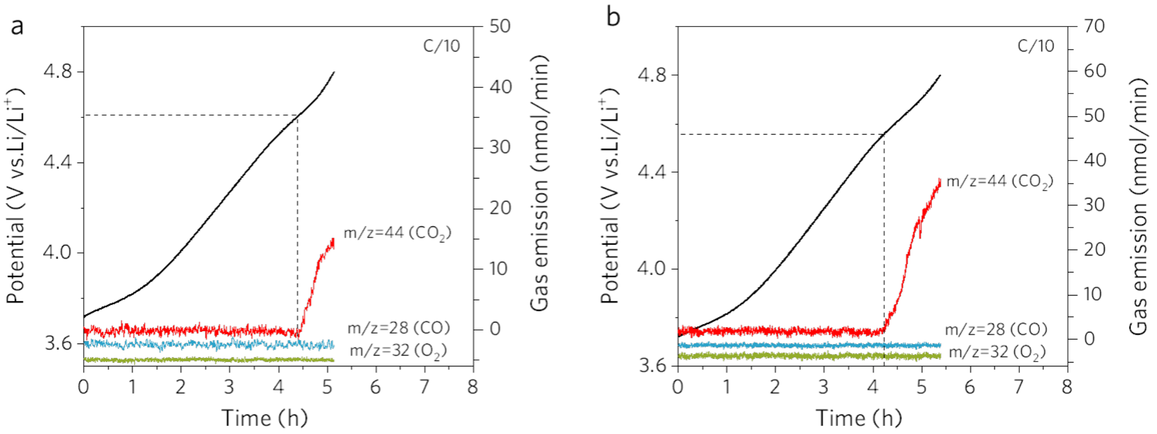

Figure S24. The OEMS for the cells employing a W-NCM111 (a) and 4M-NCM111 (b) during a charging process under a cut-off voltage of 5 V. The mass loadings of W- and 4M-NCM111 are 3.48 and 3.24 mg/cm^2. Source data are provided as a Source Data file.

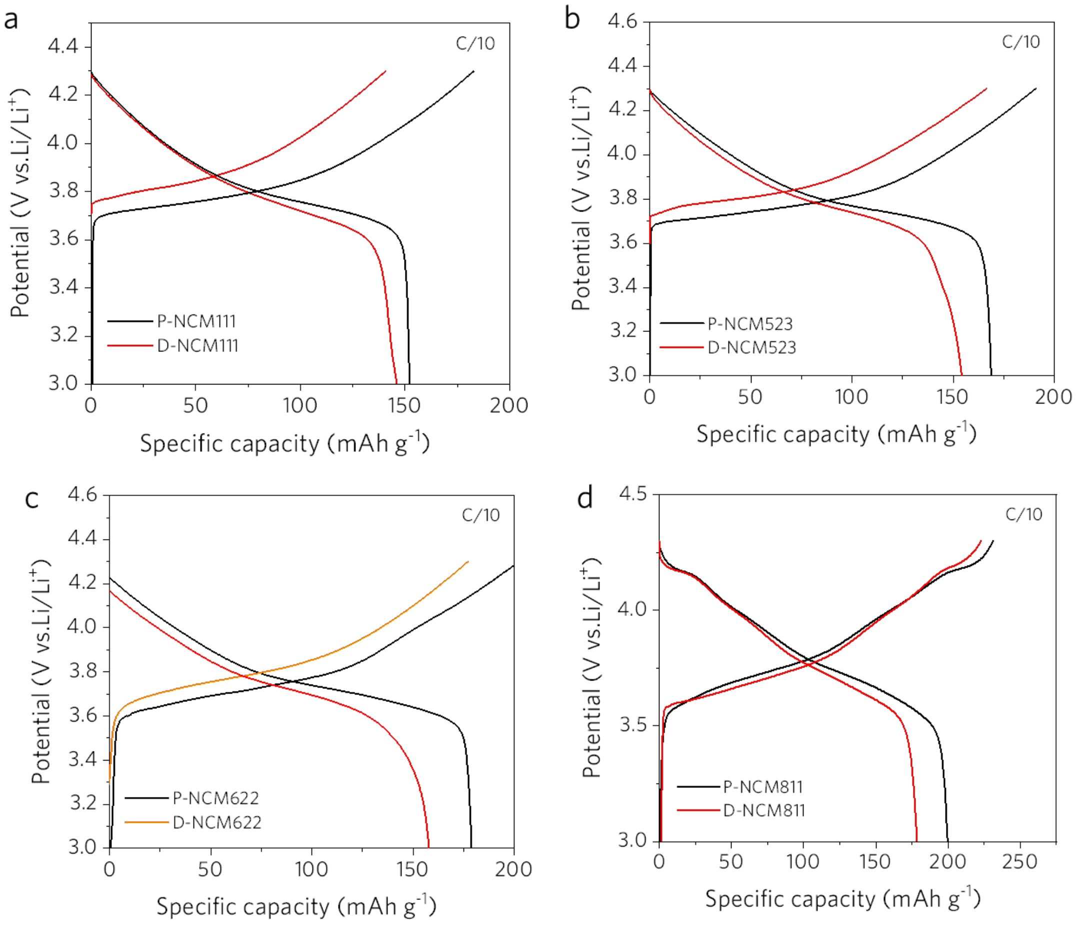

Figure S25. Charging and discharging profiles of pristine and delithiated NCM111 (a), NCM523 (b), NCM622 (c) and NCM8111 (d) in the voltage range of 3-4.3 V. 1C corresponds 150, 170, 180, 200 mA/g for NCM111, 523, 622 and 811, respectively. Source data are provided as a Source Data file.

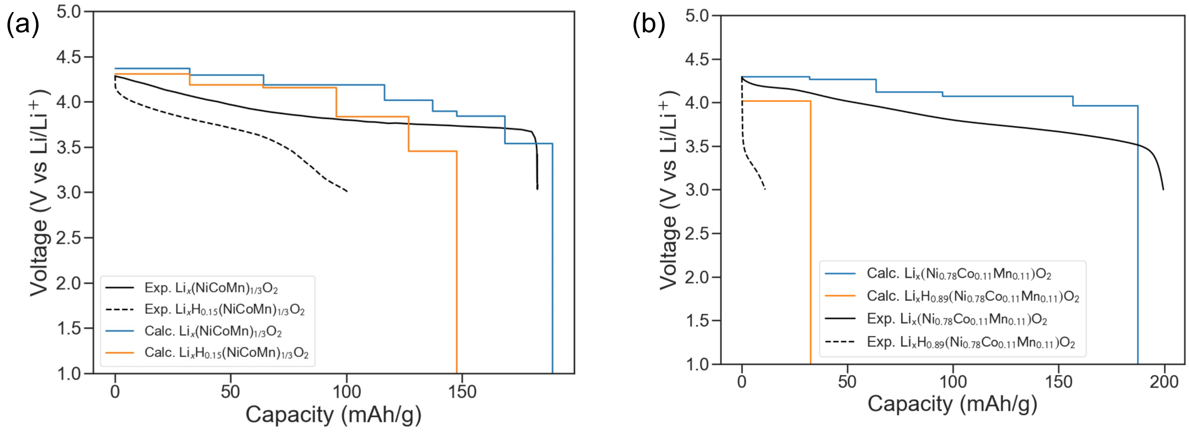

Figure S26. Calculated voltage profiles of pristine NCM111 and protonated NCM111 cathodes (a). Calculated voltage profiles of pristine NCM811 and protonated NCM811 cathodes (b). The voltage range is 3-4.3 V. The experimental observations are plotted as comparison. Source data are provided as a Source Data file.

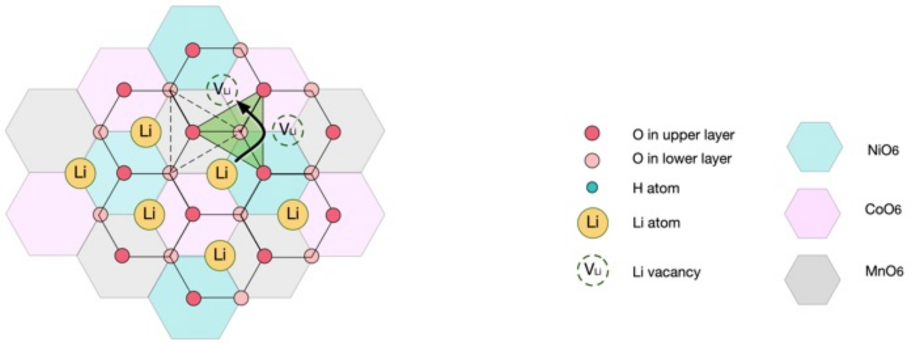

Figure S27. Schemetical illustration of Li^+ “di-vacancy” migration pathway.

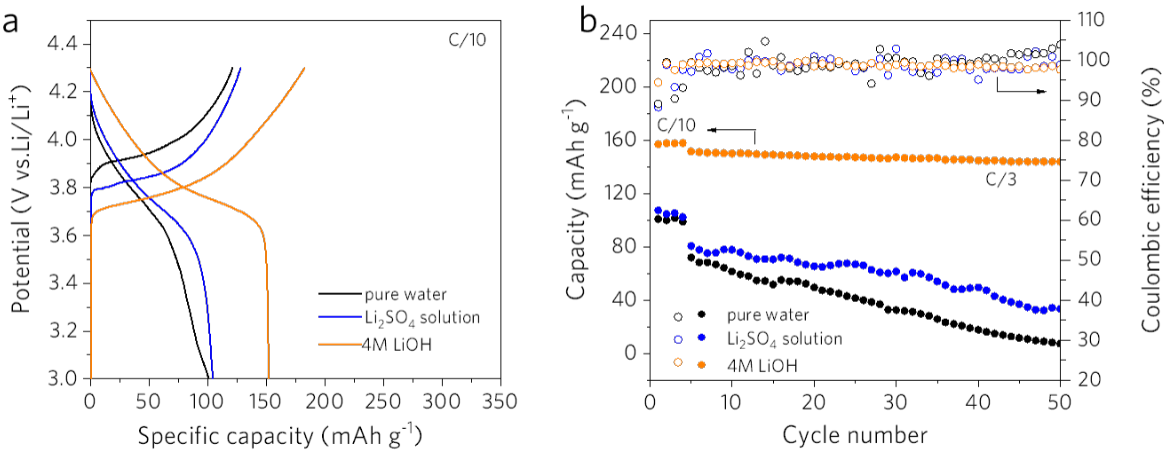

Figure S28. Charging and discharging profiles in the voltage range of 3-4.3 V (a) and cycling performance (b) of NCM111 treated with Li_2SO_4 and pure water, as well as 4M LiOH. Source data are provided as a Source Data file.

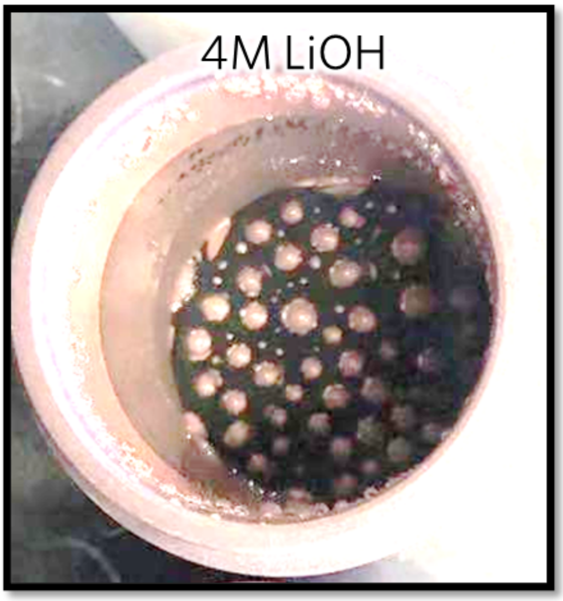

Figure S29. Digital picture of the phenomenon observed on the bottom of the reactor after treatment with 4M LiOH.

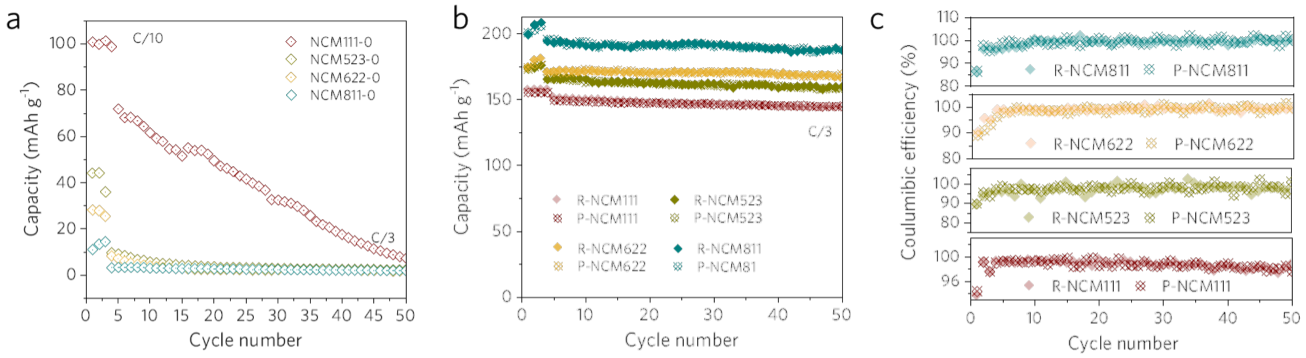

Figure S30. Cycling performance of W-NCM materials in the voltage range of 3-4.3V. (a); Comparison of the cycling performance of regenerated NCM materials (RS-NCM) and pristine materials (P-NCM). Source data are provided as a Source Data file.

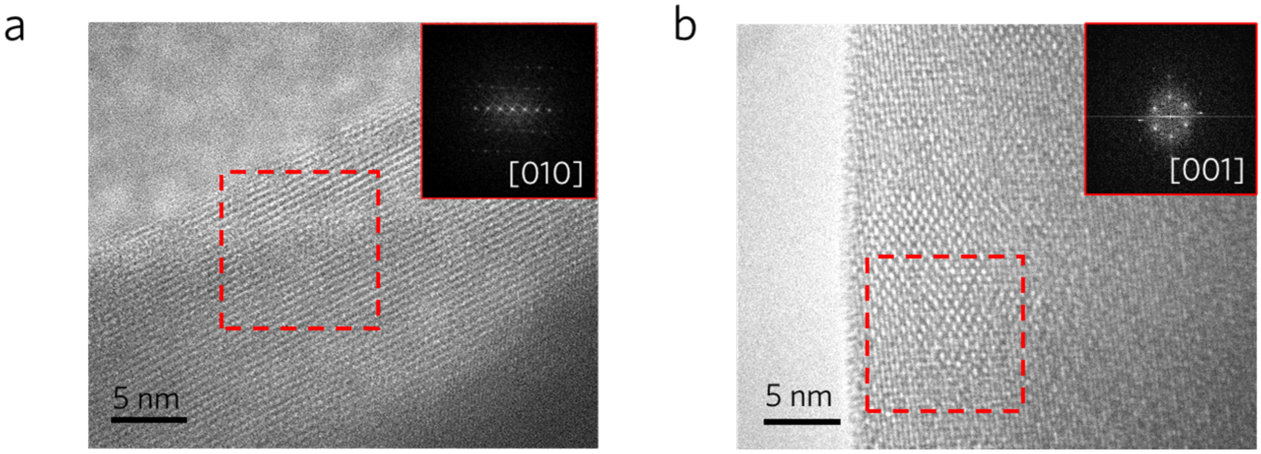

Figure S31. TEM and FFT insets of relithiated material (a) and relithiated followed by sintering material (b).

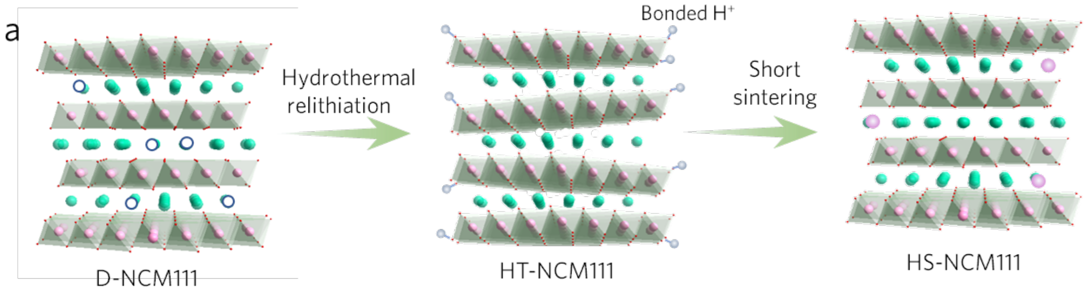

b

Hydrothermal relithiation

$$Li_{0.9}Ni_{1/3}Co_{1/3}Mn_{1/3}O_2 + (0.1\text{-}x)\ Li^+(aq) + x\ H^+(aq) \longrightarrow H_xLi_{1.0\text{-}x}Ni_{1/3}Co_{1/3}Mn_{1/3}O_2$$

Short sintering

$$H_xLi_{1.0\text{-}x}Ni_{1/3}Co_{1/3}Mn_{1/3}O_2 \longrightarrow Li_{1.0\text{-}x}Ni_{1/3}Co_{1/3}Mn_{1/3}O_{2\text{-}x/2} + x/2\ H_2O$$

$$Li_{1.0\text{-}x}Ni_{1/3}Co_{1/3}Mn_{1/3}O_{2\text{-}x/2} + x/2\ Li_2CO_3 \longrightarrow Li_{1.0}Ni_{1/3}Co_{1/3}Mn_{1/3}O_2 + x/2\ CO_2$$

Figure S32. Possible D-NCM defects repairing mechanism diagram (a) and involved chemical reactions (b).

Supporting References

1. Li, W.; Asl, H. Y.; Xie, Q.; Manthiram, A., Collapse of $LiNi_{1-x-y}Co_xMn_yO_2$ Lattice at Deep Charge Irrespective of Nickel Content in Lithium-Ion Batteries. *Journal of the American Chemical Society* **2019,** *141* (13), 5097-5101.
