# Supplementary material for: Proton-exchange induced reactivity in layered oxides for lithium-ion batteries
Source: Nat Commun. 2024 Nov 13;15:9842. doi: 10.1038/s41467-024-53731-2 (PMC11560953; doi:10.1038/s41467-024-53731-2)
Supplement: Supplementary file 2 — Description of Additional Supplementary Files [file 41467_2024_53731_MOESM2_ESM.docx]

File Name: Supplementary Data 1

Description:

All structures along with their DFT calculated energies for generating the phase diagram and voltage profiles of NCM111 in figure 2 (a) (c) (e), figure s7 (a) (b), and the evolution of lattice parameters in figure s17 (a).

File Name: Supplementary Data 2

Description:

All structures along with their DFT calculated energies for generating the phase diagram and voltage profiles of NCM811 in figure 2 (a) (c) (e), figure s7 (a) (b), and the evolution of lattice parameters in figure s17 (a).
